# Separation and Bioactive Assay of 25*R*/*S*-Spirostanol Saponin Diastereomers from *Yucca schidigera* Roezl (Mojave) Stems

**DOI:** 10.3390/molecules23102562

**Published:** 2018-10-08

**Authors:** Lu Qu, Jingya Ruan, Song Wu, Peijian Huang, Jiejing Yan, Haiyang Yu, Yi Zhang, Tao Wang

**Affiliations:** 1 Tianjin State Key Laboratory of Modern Chinese Medicine, Tianjin University of Traditional Chinese Medicine, 312 Anshanxi Road, Nankai District, Tianjin 300193, China; qululuhan88@163.com (L.Q.); Ruanjy19930919@163.com (J.R.); 17320072093@163.com (J.Y.); 2 Tianjin Key Laboratory of TCM Chemistry and Analysis, Institute of Traditional Chinese Medicine, Tianjin University of Traditional Chinese Medicine, 312 Anshanxi Road, Nankai District, Tianjin 300193, China; song201810@163.com (S.W.); hpjforever@sina.com (P.H.); hyyu@tjutcm.edu.cn (H.Y.)

**Keywords:** *Yucca schidigera* Roezl (Mojave), 25*R*/*S*-spirostanol saponin diastereomers, high performance liquid chromatography, C_30_ column, SW620 cell line, MTT

## Abstract

In order to find a simple, generic, efficient separation method for 25*R*/*S*-spirostanol saponin diastereomers, the liquid chromatographic retention behaviors of C_12_ carbonylation and C_12_ unsubstituted 25*R*/*S*-spirostanol saponin diastereomers on different stationary phases (C_8_, C_18_, C_30_ columns) and different mobile phases (MeOH-1% CH_3_COOH and CH_3_CN-1% CH_3_COOH) were investigated. A C_30_ column was firstly found to offer the highest efficiency for the separation of this kind of diastereomers than C_8_ and C_18_ columns. Meanwhile, the analysis results indicated that both CH_3_CN-1% CH_3_COOH and MeOH-1% CH_3_COOH eluate systems were selective for C_12_ unsubstituted 25*R*/*S*-spirostanol saponin diastereomers, while MeOH-1% CH_3_COOH possessed better selectivity for C_12_ carbonylation ones. Using the abovementioned analysis method, six pairs of 25*R*/*S*-spirostanol saponin diastereomers **1a**–**6a** and **1b**–**6b** from *Yucca* *schidigera* Roezl (Mojave) were isolated successfully by using HPLC on C_30_ column for the first time. Among them, three pairs were new ones, named as (25*R*)-Yucca spirostanoside E_1_ (**1a**), (25*S*)-Yucca spirostanoside E_1_ (**1b**), (25*R*)-Yucca spirostanoside E_2_ (**2a**), (25*S*)-Yucca spirostanoside E_2_ (**2b**), (25*R*)-Yucca spirostanoside E_3_ (**3a**), (25*S*)-Yucca spirostanoside E_3_ (**3b**), respectively. Moreover, **3a**, **5a**, **6a**, **3b**–**6b** showed strong inhibitory activities on the growth of SW620 cell lines with the IC_50_ values of 12.02–69.17 μM.

## 1. Introduction

*Yucca schidigera* Roezl (Mojave), belonging to the *Yucca* genus (Agavaceae family), is mainly distributed in the southwestern United States and the northern desert of Mexico. Steroidal saponins and phenolic acids are reported to be its main constituents. Because of its excellent biological activity and proven safety, it has used worldwide as a kind of additive in foods, beverages, cosmetics and feeds [1]. It is worth mentioning that extracts of *Y. schidigera* which are enriched in steroidal saponins [2], have already been developed into a commodity for a wide range of applications.

One of the main steroidal saponin aglycone types in *Y. schidigera* are the C_27_ spirostanol type steroidal saponins, which can be divided into spirostanol type (25*S*) and isospiritol type (25*R*) according to the configuration at C_25_. The successfully separation of 25*R*/*S*-spirostanol saponin diastereomers has scarecely been reported until now, although the different stereo configurations may cause complete different bioactivity and lead to unreliable results. Therefore, the successful separation of 25*R*/*S*-spirostanol saponin diastereomers and the determination of their configurations play a crucial role in further pharmacological or molecular biological research on these compounds.

The objective of this study was to establish a simple, generic, efficient separation and analysis method for the 25*R*/*S*-spirostanol saponin diastereomers. During the process, the separation ability of three stationary phase (C_8_, C_18_ and C_30_ columns) as well as two kinds of mobile phases (MeOH-1% CH_3_COOH and CH_3_CN-1% CH_3_COOH) were evaluated. As a result, the separation of 25*R*/*S*-spirostanol saponin diastereomers was accomplished by a C_30_ column, and six pairs of 25*R*/*S*-spirostanol saponin diastereomers **1a**–**6a** and **1b**–**6b** were thus obtained, the structures of which were identified by spectroscopy and chemical methods. What’s more, the potent in vitro inhibitory effects of these compounds on human colon cancer cells SW620 were assessed by the MTT method.

## 2. Results and Discussion

### 2.1. Selection of Separation Conditions for 25R/S-Spirostanol Saponin Diastereomers by HPLC

**Figure 1:**
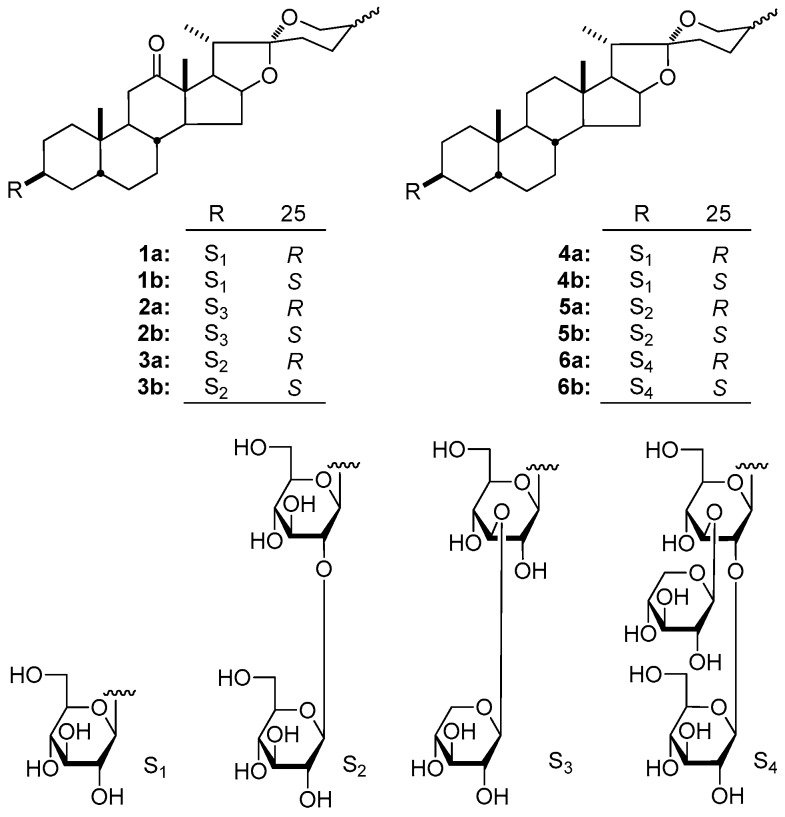
Chemical structures of the spirostanol saponins **1a**–**6a** and **1b**–**6b**.

On the basis of optimizing stationary and mobile phases, 25*R*/*S*-spirostanol saponin diastereomer mixtures **1**–**6** were isolated successfully by using the C_30_ column which possessed significant advantages for separating diastereomers. As results, six pairs of 25*R*/*S*-spirostanol saponin diastereomers were obtained, and their structures were elucidated to be new ones, (25*R*)-Yucca spirostanoside E_1_ (**1a**), (25*S*)-Yucca spirostanoside E_1_ (**1b**), (25*R*)-Yucca spirostanoside E_2_ (**2a**), (25*S*)-Yucca spirostanoside E_2_ (**2b**), (25*R*)-Yucca spirostanoside E_3_ (**3a**), (25*S*)-Yucca spirostanoside E_3_ (**3b**), and known ones, (25*R*)-5β-spirostan-3β-ol 3-*O*-β-d-glucopyranoside (**4a**) [3], asparagoside A (**4b**) [3], 25(*R*)-schidigera-saponin D5 (**5a**) [4], 25(*S*)-schidigera-saponin D5 (**5b**) [4], 25(*R*)-schidigera-saponin D1 (**6a**) [4], 25(*S*)-schidigera-saponin D1 (**6b**) [4] (Figure 1), respectively.

The 25*R*/*S*-spirostanol saponin diastereomers mentioned above could be divided into two classes according to their aglycone types: C_12_ carbonylated (compounds **1a**–**3a**, **1b**–**3b**) and C_12_ unsubstituted (compounds **4a**–**6a**, **4b**–**6b**) 25*R*/*S*-spirostanol saponins. In the course of comparing separation ability of three stationary phase (C_8_, C_18_ and C_30_ columns) as well as two kinds of mobile phases (MeOH-1% CH_3_COOH and CH_3_CN-1% CH_3_COOH) [using an evaporating light scattering detector (ELSD) detector] for the abovementioned 25*R*/*S*-spirostanol saponin diastereomers, we found that the liquid chromatography retention behaviors of two kinds of aglycone type 25*R*/*S*-spirostanol saponin diastereomers were different, and the specific rules are summed up in the following subsections.

#### 2.1.1. General Rules and Characteristics HPLC Analysis for C_12_ Carbonylation 25*R*/*S*-Spirostanol Saponin Diastereomers **1a**–**3a**, **1b**–**3b**

**Figure 2:**
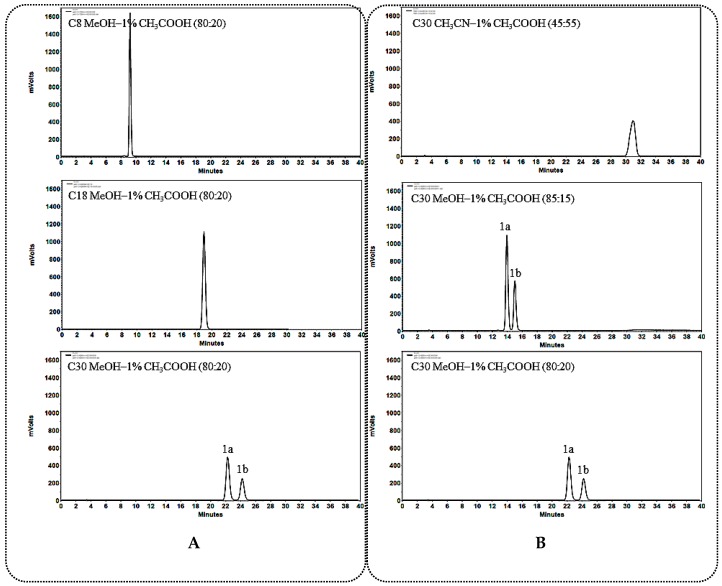
(**A**) Chromatograms of **1a** and **1b** separated on C_8_, C_18_, C_30_ columns; (**B**) Chromatograms of **1a** and **1b** separated on the C_30_ column in different solvent system.

**Figure 3:**
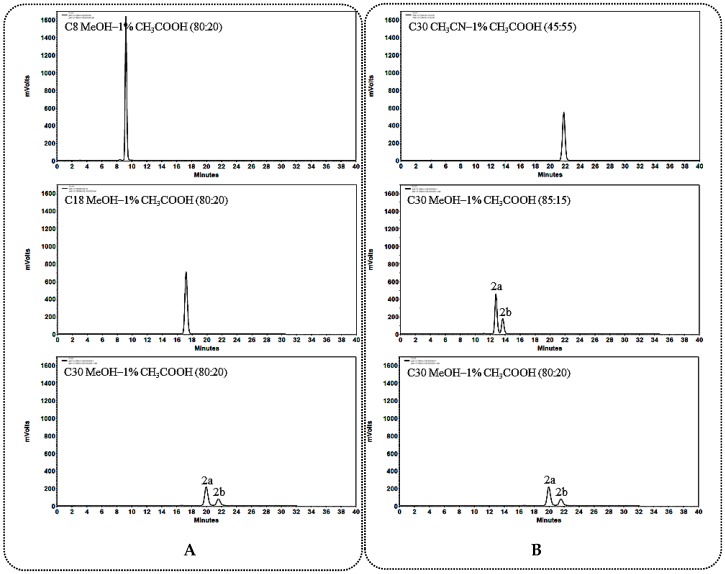
(**A**) Chromatograms of **2a** and **2b** separated on C_8_, C_18_, C_30_ columns; (**B**) Chromatograms of **2a** and **2b** separated on the C_30_ column in different solvent systems.

**Figure 4:**
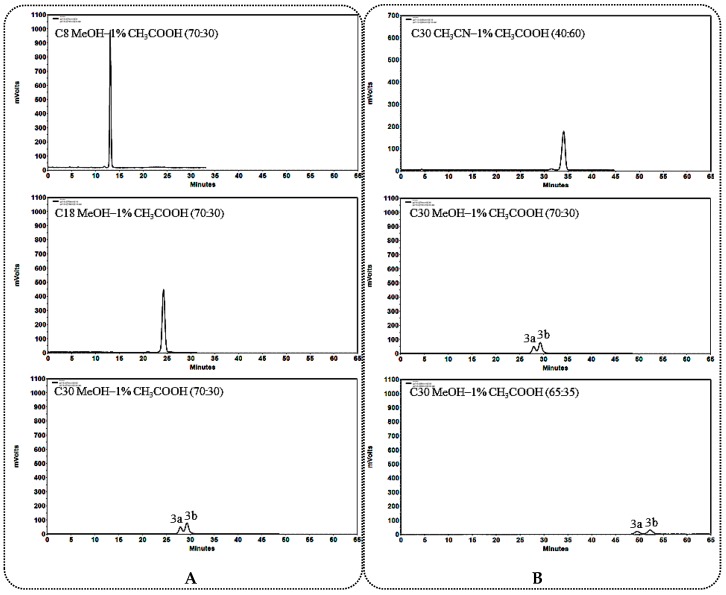
(**A**) Chromatograms of **3a** and **3b** separated on C_8_, C_18_, C_30_ columns; (**B**) Chromatograms of **3a** and **3b** separated on the C_30_ column in different solvent systems.

When using a C_30_ column as stationary phase, better separation effect could be obtained on C_12_ carbonylated 25*R*/*S*-spirostanol saponin diastereomers (Figure 2A, Figure 3A and Figure 4A). During the process of optimizing the separation conditions on the C_30_ column, MeOH-1% CH_3_COOH was found to possess better selectivity. Moreover, the longer for the retention time (*t*_R_), the better resolution (Figure 2B, Figure 3B and Figure 4B) was obtained. On the other hand, we found that the *t*_R_ of 25*R*-spirostanol saponins **1a**–**3a** was always shorter than that of 25*S* ones **1b**–**3b** with the MeOH-1% CH_3_COOH eluate system, which was not related to the type or number of substituted sugars.

#### 2.1.2. General Rules and Characteristics HPLC Analysis for C_12_ Unsubstituted 25*R*/*S*-Spirostanol Saponin Diastereomers **4a**–**6a**, **4b**–**6b**

**Figure 5:**
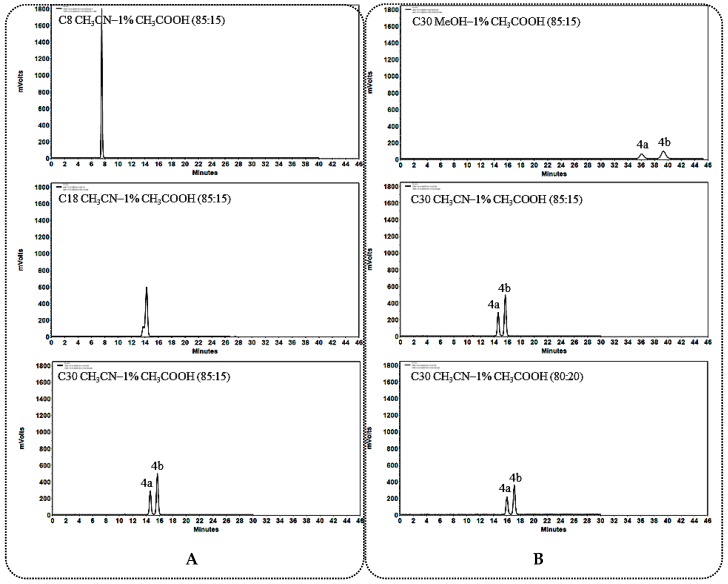
(**A**) Chromatograms of **4a** and **4b** separated on C_8_, C_18_, C_30_ column; (**B**) Chromatograms of **4a** and **4b** separated on the C_30_ column in different solvent systems.

**Figure 6:**
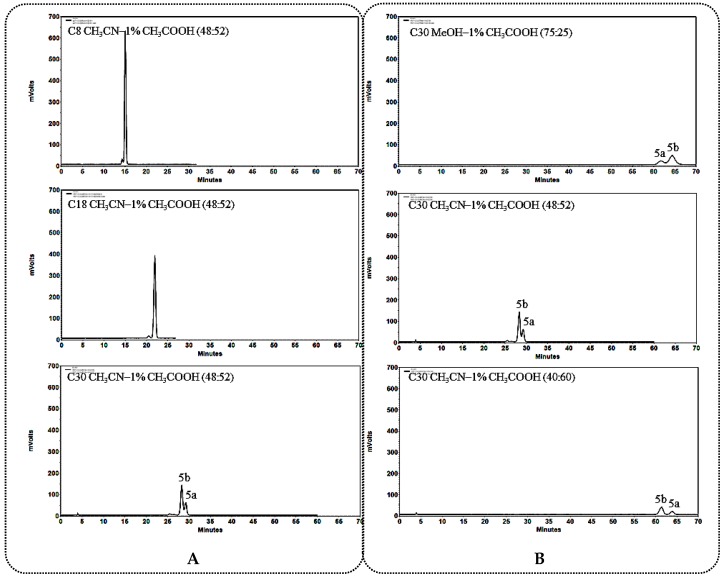
(**A**) Chromatograms of **5a** and **5b** separated on C_8_, C_18_, C_30_ column; (**B**) Chromatograms of **5a** and **5b** separated on the C_30_ column in different solvent systems.

**Figure 7:**
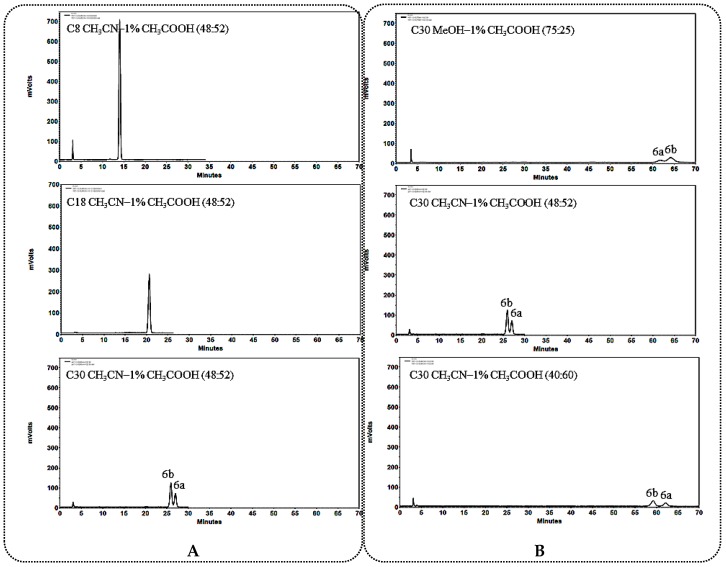
(**A**) Chromatograms of **6a** and **6b** separated on C_8_, C_18_, C_30_ column; (**B**) Chromatograms of **6a** and **6b** separated on the C_30_ column in different solvent systems.

The C_30_ column was also found to be more suitable for the separation of C_12_ unsubstituted 25*R*/*S*-spirostanol saponin diastereomers (Figure 5A, Figure 6A and Figure 7A) than the C_8_ and C_18_ columns. The difference from the C_12_ carbonylated 25*R*/*S*-spirostanol saponin diastereomers was that both the CH_3_CN-1% CH_3_COOH and MeOH-1% CH_3_COOH eluate systems were selective to this type of compounds, while CH_3_CN-1% CH_3_COOH system could guarantee the resolution in shorter *t*_R_ (Figure 5B, Figure 6B and Figure 7B).

In addition, the *t*_R_ of monosaccharide substituted 25*R*-spirostanol saponin **4a** was shorter than that of 25*S*-spirostanol saponin **4b** whether in MeOH-1% CH_3_COOH or in CH_3_CN-1% CH_3_COOH system. As the number of glycosyl groups increase (compounds **5a**, **5b**, **6a**, **6b**), the *t*_R_ of 25*R*-spirostanol saponin was always shorter than that of 25*S*-spirostanol saponin (**5a** vs. **5b**, **6a** vs. **6b**) in MeOH-1% CH_3_COOH eluate system, a phenomenon in contrast in the CH_3_CN-1% CH_3_COOH eluate system case.

### 2.2. Structure Identification for 25R/S-Spirostanol Saponin Diastereomers

**Figure 8:**
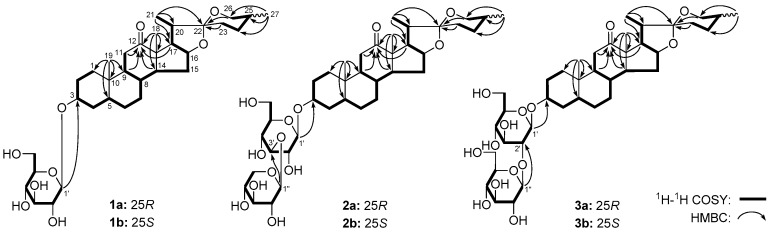
The main ^1^H-^1^H COSY and HMBC correlations of **1a**–**3a** and **1b**–**3b**.

Both (25*R*)-Yucca spirostanoside E_1_ (**1a**) and (25*S*)-Yucca spirostanoside E_1_ (**1b**) were isolated as white powders with negative optical rotation [([α]${}_{D}^{25}$ − 11.2°, MeOH) for **1a**, ([α]${}_{D}^{25}$ − 6.6°, MeOH) for **1b**]. Their molecular formulae were deduced to be C_33_H_52_O_9_ by the positive-ion HRESI-MS analysis (*m*/*z* 593.3705 [M + H]^+^ for **1a**, and 593.3703 [M + H]^+^ for **1b**, both calcd. for C_33_H_53_O_9_, 593.3684). Treatment with 1 M hydrochloric acid (HCl) liberated d-glucose, which was identified by HPLC analysis using an optical rotation detector [5]. Thirty-three carbon signals were displayed in their ^13^C-NMR (Table 1, C_5_D_5_N) spectrum. Besides the carbon signals represented by d-glucose, the other twenty-seven ones, especially the quaternary carbon signal at δ_C_ 109.3 (**1a**)/109.8 (**1b**) indicated that they were spirostane-type steroid saponins. The ^1^H-NMR spectrum suggested the presence of four methyls [δ 0.70 (3H, d, *J* = 6.0 Hz, H_3_-27), 0.85, 1.09 (3H each, both s, H_3_-19 and 18), 1.37 (3H, d, *J* = 6.5 Hz, H_3_-21) for **1a**; δ 1.07 (3H, d, *J* = 6.5 Hz, H_3_-27), 0.84, 1.08 (3H each, both s, H_3_-19 and 18), 1.37 (3H, d, *J* = 7.0 Hz, H_3_-21) for **1b**], two methines bearing an oxygen function [δ 4.32 (1H, m, H-3), 4.55 (1H, q like, ca. *J* = 8 Hz, H-16) for **1a**; δ 4.32 (1H, m, H-3), 4.52 (1H, q like, ca. *J* = 7 Hz, H-16) for **1b**], one oxygenated methene {[δ 3.50 (1H, dd, *J* = 10.5, 10.5 Hz), 3.60 (1H, dd, *J* = 4.0, 10.5 Hz), H_2_-26] for **1a**; [δ 3.38 (1H, br. d, ca. *J* = 11 Hz), 4.06 (1H, dd, *J* = 2.5, 11.0 Hz), H_2_-26] for **1b**} and one β-d-glucopyranosyl [δ 4.93 (1H, d, *J* = 7.5 Hz, H-1′) for **1a**; δ 4.92 (1H, d, *J* = 7.5 Hz, H-1′) for **1b**] in their aglycones. The existence of carbonyl was clarified by the ^13^C-NMR signal at δ_C_ 213.0 (C-12) (**1a**/**1b**). The ^1^H-^1^H COSY spectra of **1a** and **1b** suggested the presence of the three partial structures written in bold lines in Figure 8. The planar structure of their aglycons were determined to be spirostan-3-ol-12-one based on the key HMBC correlations from H_2_-11, H-14, 17 to C-12; H_3_-18 to C-12–14, 17; H_3_-19 to C-1, 5, 9, 10; H_3_-21 to C-17, 20, 22; H_2_-23, 26 to C-22; H_3_-27 to C-24–26. Moreover, the β-d-glucopyranosyl was determined to link at C-3 position of aglycone by the long-range correlation from H-1′ to C-3 observed in the HMBC experiment. Their ^1^H- and ^13^C-NMR data for the protons and carbons in A–E ring were identical to those of Yucca spirostanoside C_1_ [5], and the configuration of A–E ring was determined. Comparing the proton chemical shifts, we found CH_3_-27 of **1a** (δ 0.70) displayed signal at the higher field than that of **1b** (δ 1.07); what’s more, there was a smaller difference between the two protons of CH_2_-26 of **1a** (∆δ_a,b_ = 0.10 ppm) than that of **1b** (∆δ_a,b_ = 0.68 ppm). According to the rules summarized by Boll et al. [6] and Schreiber et al. [7], the absolute configuration of C-25 was elucidated to be *R* and *S* for **1a** and **1b**, respectively. On the other hand, the comparision resultsof their ^13^C-NMR data for F ring (C-22–26) and C-27 [δ 17.3 (C-27), 29.2 (C-24), 30.5 (C-25), 31.8 (C-23), 66.9 (C-26), 109.3 (C-22) for **1a**; δ 16.3 (C-27), 26.1 (C-24), 26.4 (C-23), 27.5 (C-25), 65.2 (C-26), 109.8 (C-2) for **1b**] with those of (25*R*)-5β-spirostan [δ 17.1 (C-27), 28.8 (C-24), 30.3 (C-25), 31.4 (C-23), 66.8 (C-26), 109.2 (C-22)] and (25*S*)-5β-spirostan [δ 16.1 (C-27), 25.8 (C-24), 26.0 (C-25), 27.1 (C-23), 65.2 (C-26), 109.7 (C-22)] [8], clarified the absolute configuration of C-25 furtherly. On the basis of above mentioned evidence, the structure of **1a** and **1b** was elucidated to be (25*R*)-5β-spirostan-3β-ol-12-one 3-*O*-β-d-gluco- pyranoside and (25*S*)-5β-spirostan-3β-ol-12-one 3-*O*-β-d-glucopyranoside, respectively.

The Q-TOF-ESI-MS analysis results indicated that (25*R*)-Yucca spirostanoside E_2_ (**2a**) and (25*S*)-Yucca spirostanoside E_2_ (**2b**), (25*R*)-Yucca spirostanoside E_3_ (**3a**) and (25*S*)-Yucca spirostanoside E_3_ (**3b**) had the same molecular formula, C_38_H_60_O_13_ and C_39_H_62_O_14_, respectively. Acid hydrolysis reaction experiments proved that **2a** and **2b** contained d-glucose and d-xylose [5], while only d-glucose existed in **3a** and **3b**. Their ^1^H-, ^13^C- (Table 1, C_5_D_5_N) and 2D- (^1^H-^1^H COSY, HSQC, HMBC) NMR spectra suggested that the aglycons of compounds **2a** and **3a**, **2b** and **3b** were (25*R*)-5β-spirostan-3β-ol-12-one, (25*S*)-5β-spirostan-3β-ol-12-one, respectively [6,7,8]. Meanwhile, the long-rang correlations from H-1′ to C-3; H-1″ to C-3′ could be observed in the HMBC spectra of compounds **2a** and **2b**; and in the HMBC spectra of **3a** and **3b**, the correlations from H-1′ to C-3; H-1″ to C-2′ could be observed. Consequently, the structures of **2a**, **2b**, **3a**, and **3b** were elucidated.

The structures of known compounds **4a**–**b** [3], **5a**–**b** [4] and **6a**–**b** [4] were identified by comparing their ^1^H-, ^13^C-NMR data with references.

### 2.3. Inhibitory Activities on the Growth of SW620 Cell Lines Study of Extract, Fractions, and Compounds Obtained from Y. schidigera

The inhibitory effects of *Y. schidigera* 70% EtOH extract, *Y. schidigera* 95% EtOH eluate, *Y. schidigera* H_2_O eluate, as well as 25*R*/*S*-spirostanol saponin diastereomers **1a**–**6a**, **1b**–**6b** on the growth of SW620 cell lines were measured by the MTT method. As the results in Table 2 show, *Y. schidigera* 70% EtOH extract and *Y. schidigera* 95% EtOH eluate displayed IC_50_ values as 85.20 and 93.04 μg/mL, respectively. Meanwhile, compound **6b** exhibited strong activity with IC_50_ value of 12.02 μM comparable with that of the positive control 5-fluorouracil (5-FU, IC_50_ 10.00 μM), and **3a**, **5a**, **6a**, **3b**–**5b** showed the IC_50_ values of 29.81–69.17 μM. Moreover, through the summary of structure-activity relationships of 25*R*/*S*-spirostanol saponin diastereomers **1a**–**6a**, **1b**–**6b**, it could be found that the configuration of C_25_ had significant influence of the inhibitory activities towards SW620 cells. For C_12_ unsubstituted 25*R*/*S*-spirostanol saponin diastereomers, the bioactivity of 25*S*-spirostanol saponins was stronger than that of 25*R*-ones (**4b** vs. **4a**; **5b** vs. **5a**; **6b** vs. **6a**); however, it was exactly opposite for C_12_ carbonylation 25*R*/*S*-spirostanol saponin diastereomers (**3b** vs. **3a**). What’s more, the numbers of substituted glycosyls also affected their activities. For example, as the substituted glycosyls increased, C_12_ unsubstituted 25*R*/*S*-spirostanol saponin diastereomers showed stronger inhibitory effects on SW620 cells (**6b** vs. **5b** vs. **4b**; **6a** vs. **5a** vs. **3a**).

## 3. Materials and Methods

### 3.1. General Information

The following instruments were used to measure physical data: IR spectra were determined on a 640-IR FT-IR spectrophotometer (Varian Australia Pty Ltd., Mulgrave, Australia). Optical rotations were run on an Autopol^®^ IV automatic polarimeter (l = 50 mm, Rudolph Research Analytical, Hackettstown, NJ, USA). NMR spectra were obtained on a Bruker 500 MHz NMR spectrometer (Bruker BioSpin AG Industriestrasse 26 CH-8117, Fällanden, Switzerland) at 500 MHz for ^1^H- and 125 MHz for ^13^C-NMR (internal standard: TMS). Positive-ion HRESI-TOF-MS were recorded on an Agilent Technologies 6520 Accurate-Mass Q-Tof LC/MS spectrometer (Agilent Corp., Santa Clara, CA, USA). High performance liquid chromatography (HPLC) analyses were performed on an Agilent 1260 Infinity system (Agilent Technologies Inc.) equipped with ELSD (Alltech 2000 ES, Chengdu, China).

Column chromatographies (CC) were performed on macroporous resin D101 (Haiguang Chemical Co., Ltd., Tianjin, China), Silica gel (74–149 µm, Qingdao Haiyang Chemical Co., Ltd., Qingdao, China), and ODS (40–63 μm, YMC Co., Ltd., Tokyo, Japan). High performance liquid chromatography (HPLC) columns: Cosmosil 5C_18_-MS-II (4.6 mm i.d. × 250 mm, Nacalai Tesque, Inc., Kyoto, Japan), Cosmosil C_8_-MS (4.6 mm i.d. × 250 mm, Nacalai Tesque, Inc.), Cosmosil PBr (4.6 mm i.d. × 250 mm, Nacalai Tesque, Inc.), Wacopak Navi C_30_-5 (4.6 mm i.d. × 250 mm, Wako Pure Chemical Industries, Ltd., Osaka, Japan) were used to analyze the mixture. Preparative high performance liquid chromatography (PHPLC) columns: Cosmosil 5C_18_-MS-II (20 mm i.d. × 250 mm, Nacalai Tesque, Inc.), Wacopak Navi C_30_-5 (7.5 mm i.d. × 250 mm, Wako Pure Chemical Industries, Ltd.), and Cosmosil PBr (20 mm i.d. × 250 mm, Nacalai Tesque, Inc.) were used to separate the constituents.

### 3.2. Plant Material

The stems of *Y. schidigera* were collected from National City, in southwest Califonia, USA, and identified by Dr. Li Tianxiang (The Hall of TCM Specimens, Tianjin University of TCM, Tianjin, China). The voucher specimen was deposited at the Academy of Traditional Chinese Medicine of Tianjin University of TCM (No. 20160301).

### 3.3. Extraction and Isolation

#### 3.3.1. Extraction and Isolation of 25*R*/*S*-Spirostanol Saponin Diastereomer Mixtures **1**–**6**

The dried stems of *Y. schidigera* (5.0 kg) were refluxed with 70% ethanol-water for three times. Evaporation of the solvent under pressure provided a 70% ethanol-water (800.0 g). The residue (700.0 g) was dissolved in H_2_O, and subjected to D101 CC (H_2_O → 95% EtOH) to afford H_2_O (380.4 g) and 95% EtOH (310.1 g) eluates, respectively.

The 95% EtOH eluate (200.0 g) was subjected to silica gel CC [CH2Cl2 → CH2Cl2-MeOH (100:1 → 100:3 → 100:7 → 5:1 → 3:1 → 2:1, *v*/*v*) → MeOH] to afford thirteen fractions (Fr. 1–Fr. 13). Fraction 6 (12.0 g) was separated by ODS CC [MeOH-H2O (30:70 → 40:60 → 50:50 → 60:40 → 70:30 → 80:20 → 100:0, *v*/*v*)], and fourteen fractions (Fr. 6-1–Fr. 6-14) were obtained. Fraction 6-12 (800.9 mg) was isolated by PHPLC [MeOH-1% CH_3_COOH (75:25, *v*/*v*), Cosmosil 5C_18_-MS-II column] to yield mixtures **1** and **2**. Fraction 6-13 (1.2 g) was subjected to silica gel CC [CH_2_Cl_2_-MeOH (100:3 → 100:5 → 100:7) → MeOH, *v*/*v*] to produce nine fractions (Fr. 6-13-1–Fr. 6-13-9). Fraction 6-13-3 (446.3 mg) was isolated by PHPLC [MeOH-1% CH_3_COOH (90:10, *v*/*v*), Cosmosil 5C_18_-MS-II column] to provide mixture **4**. Fraction 7 (10.0 g) was subjected to PHPLC [MeOH-1% CH_3_COOH (80:20, *v*/*v*), Cosmosil 5C_18_-MS-II column] to produce thirteen fractions (Fr. 7-1–Fr. 7-13). Fraction 7-5 (712.6 mg) was separated by PHPLC [CH_3_CN-1% CH_3_COOH (40:60, *v*/*v*), Cosmosil 5C_18_-MS-II column] and PHPLC [MeOH-1% CH_3_COOH (70:30, *v*/*v*), Cosmosil 5C_18_-MS-II column] to obtain mixture **3**. Fraction 7-12 (984.6 mg) was separated by PHPLC [MeOH-1% CH_3_COOH (95:5, *v*/*v*), Cosmosil PBr column] to give mixtures **5** and **6**.

#### 3.3.2. Extraction and Isolation of 25*R*/*S*-Spirostanol Saponin Diastereomers **1a**–**6a**, **1b**–**6b** by Using C_30_ Column

Mixture **1** (190.3 mg) was purified by PHPLC [MeOH-1% CH_3_COOH (85:15, *v*/*v*)] to give (25*R*)-Yucca spirostanoside E_1_ (**1a**, 37.8 mg) and (25*S*)-Yucca spirostanoside E_1_ (**1b**, 23.0 mg). Mixture **2** (160.7 mg) was separated by using the same method as that for mixture **1** to gain (25*R*)-Yucca spirostanoside E_2_ (**2a**, 47.5 mg) and (25*S*)-Yucca spirostanoside E_2_ (**2b**, 32.0 mg). Mixture **3** (15.0 mg) was separated by HPLC [MeOH-1% CH_3_COOH (70:30, *v*/*v*)] to yield (25*R*)-Yucca spirostanoside E_3_ (**3a**, 3.8 mg) and (25*S*)-Yucca spirostanoside E_3_ (**3b**, 7.2 mg). Mixture **4** was (180.0 mg) isolated by PHPLC [CH_3_CN-1% CH_3_COOH (80:20, *v*/*v*)] to provide (25*R*)-5β-spirostan-3β-ol 3-*O*-β-d-gluco- pyranoside (**4a**, 10.0 mg) and asparagoside A (**4b**, 36.5 mg). Mixture **5** (10.0 mg) was purified by HPLC [CH_3_CN-1% CH_3_COOH (40:60, *v*/*v*)] to give 25(*R*)-schidigera-saponin D5 (**5a**, 2.0 mg) and 25(*S*)-schidigera-saponin D5 (**5b**, 4.2 mg). Using the same HPLC condition, 25(*R*)-schidigera-saponin D1 (**6a**, 5.5 mg) and 25(*S*)-schidigera-saponin D1 (**6b**, 10.0 mg) were obtained.

*(25R)-Yucca spirostanoside E_1_* (**1a**): White powder; [α]${}_{D}^{25}$ − 11.2° (*c =* 0.97, MeOH); IR ν_max_ (KBr) cm^−1^: 3395, 2929, 2871, 1705, 1454, 1379, 1345, 1244, 1161, 1074, 1027, 985, 921, 897, 867. ^1^H-NMR (C_5_D_5_N, 500 MHz): δ 0.70 (3H, d, *J* = 6.0 Hz, H_3_-27), 0.85 (3H, s, H_3_-19), 0.97, 1.33 (1H each, both m, H_2_-7), 1.09 (3H, s, H_3_-18), 1.11, 1.76 (1H each, both m, H_2_-6), [1.28 (1H, m), 1.73 (1H, m, overlapped), H_2_-1], 1.37 (3H, d, *J* = 6.5 Hz, H_3_-21), 1.42, 1.86 (1H each, both m, H_2_-2), 1.47 (1H, m, H-14), 1.57 (2H, m, overlapped, H_2_-24), 1.58 (1H, m, overlapped, H-25), [1.61 (1H, m, overlapped), 2.14 (1H, ddd, *J* = 5.5, 8.0, 11.5 Hz), H_2_-15], 1.63, 1.71 (1H each, both m, H_2_-23), 1.73 (2H, m, overlapped, H_2_-4), 1.75 (1H, m, overlapped, H-9), 1.83 (1H, m, H-8), 1.94 (1H, quin, *J* = 6.5 Hz, H-20), 2.08 (1H, m, H-5), [2.21 (1H, dd, *J* = 4.5, 14.0 Hz), 2.37 (1H, dd, *J* = 14.0, 14.0 Hz), H_2_-11], 2.82 (1H, dd, *J* = 6.5, 8.5 Hz, H-17), [3.50 (1H, dd, *J* = 10.5, 10.5 Hz), 3.60 (1H, dd, *J* = 4.0, 10.5 Hz), H_2_-26], 3.95 (1H, m, H-5′), 4.05 (1H, dd, *J* = 7.5, 8.5 Hz, H-2′), 4.27 (1H, m, overlapped, H-3′), 4.27 (1H, m, overlapped, H-4′), 4.32 (1H, m, H-3), [4.40 (1H, dd, *J* = 5.0, 11.5 Hz), 4.54 (1H, m, overlapped), H_2_-6′], 4.55 (1H, q like, ca. *J* = 8 Hz, H-16), 4.93 (1H, d, *J* = 7.5 Hz, H-1′); ^13^C-NMR (C_5_D_5_N, 125 MHz) spectroscopy data: see Table 1. HRESI-TOF-MS: Positive-ion mode *m*/*z* 593.3705 [M + H]^+^ (calcd. for C_33_H_53_O_9_, 593.3684).

*(25S)-Yucca spirostanoside E_1_* (**1b**): White powder; [α]${}_{D}^{25}$ − 6.6° (*c =* 1.06, MeOH); IR ν_max_ (KBr) cm^−1^: 3391, 2930, 2874, 1704, 1454, 1378, 1345, 1269, 1170, 1070, 1025, 988, 920, 897, 849. ^1^H-NMR (C_5_D_5_N, 500 MHz): δ 0.84 (3H, s, H_3_-19), [0.96 (1H, m), 1.35 (1H, m, overlapped), H_2_-7] 1.08 (3H, s, H_3_-18), 1.07 (3H, d, *J* = 6.5 Hz, H_3_-27), [1.11 (1H, m), 1.76 (m, overlapped), H_2_-6], [1.27 (1H, m), 1.72 (1H, m, overlapped), H_2_-1], [1.33 (1H, m, overlapped), 1.91 (1H, m, overlapped), H_2_-23], [1.33 (1H, m, overlapped), 2.13 (1H, m, overlapped), H_2_-24], 1.37 (3H, d, *J* = 7.0 Hz, H_3_-21), [1.41 (1H, m, overlapped), 1.86 (1H, m), H_2_-2], 1.45 (1H, m, H-14), 1.59 (1H, m, overlapped, H-25), [1.59 (1H, m, overlapped), 2.12 (1H, m, overlapped), H_2_-15], 1.72 (m, overlapped, H_2_-4), 1.75 (1H, m, overlapped, H-9), 1.82 (1H, m, H-8), 1.88 (1H, m, overlapped, H-20), 2.08 (1H, m, H-5), [2.20 (1H, dd, *J* = 4.5, 14.5 Hz), 2.36 (1H, dd, *J* = 14.5, 14.5 Hz), H_2_-11], 2.79 (1H, dd, *J* = 6.5, 8.5 Hz, H-17), [3.38 (1H, br. d, ca. *J* = 11 Hz), 4.06 (1H, dd, *J* = 2.5, 11.0 Hz), H_2_-26], 3.94 (1H, m, H-5′), 4.05 (1H, dd, *J* = 7.5, 8.0 Hz, H-2′), 4.25 (1H, m, overlapped, H-3′), 4.25 (1H, m, overlapped, H-4′), 4.32 (1H, m, H-3), [4.40 (1H, dd, *J* = 5.0, 11.5 Hz), 4.54 (1H, dd, *J* = 2.5, 11.5), H_2_-6′], 4.52 (1H, q like, ca. *J* = 7 Hz, H-16), 4.92 (1H, d, *J* = 7.5 Hz, H-1′); ^13^C-NMR (C_5_D_5_N, 125 MHz) spectroscopy data: see Table 1. HRESI-TOF-MS: Positive-ion mode *m*/*z* 593.3703 [M + H]^+^ (calcd. for C_33_H_53_O_9_, 593.3684).

*(25R)-Yucca spirostanoside E_2_* (**2a**): White powder; [α]${}_{D}^{25}$ − 0.29° (*c =* 0.67, MeOH); IR ν_max_ (KBr) cm^−1^: 3427, 2928, 2869, 1707, 1454, 1375, 1240, 1162, 1074, 1040, 984, 922, 897, 866. ^1^H-NMR (C_5_D_5_N, 500 MHz): δ 0.70 (3H, d, *J* = 6.0 Hz, H_3_-27), 0.87 (3H, s, H_3_-19), 1.10 (3H, s, H_3_-18), 1.16, 1.81 (1H each, both m, H_2_-6), [1.28 (1H, m), 1.71 (1H, m, overlapped), H_2_-1], 1.37 (3H, d, *J* = 7.0 Hz, H_3_-21), [1.37 (1H, m, overlapped), 1.81 (1H, m), H_2_-2)], 1.48 (1H, m, H-14), 1.57 (2H, m, overlapped, H_2_-24), 1.58 (1H, m, overlapped, H-25), [1.60 (1H, m, overlapped), 2.15 (1H, ddd, *J* = 6.5, 7.5, 13.5 Hz), H_2_-15], [1.64 (1H, m), 1.71 (1H, m, overlapped), H_2_-23], 1.73 (2H, m, H_2_-4), 1.76 (1H, m, H-9), 1.84 (1H, m, H-8), 1.94 (1H, quin, *J* = 7.0 Hz, H-20), 2.08 (1H, m, H-5), [2.21 (1H, dd, *J* = 5.0, 14.5 Hz), 2.38 (1H, dd, *J* = 14.5, 14.5 Hz), H_2_-11], 2.82 (1H, dd, *J* = 6.5, 8.5 Hz, H-17), [3.50 (1H, dd, *J* = 10.5, 10.5 Hz), 3.60 (1H, dd, *J* = 3.5, 10.5 Hz), H_2_-26], [3.69 (1H, dd, *J* = 11.0, 11.0 Hz), 4.31 (1H, m, overlapped), H_2_-5″], 3.90 (1H, m, H-5′), 4.02 (1H, dd, *J* = 7.5, 8.0 Hz, H-2″), 4.07 (1H, dd, *J* = 8.0, 8.5 Hz, H-2′), 4.14 (1H, dd, *J* = 8.0, 9.0 Hz, H-3″), 4.16 (1H, m, H-4″), 4.18 (1H, dd, *J* = 9.0, 9.5 Hz, H-4′), 4.20 (1H, m, overlapped, H-3), 4.25 (1H, dd, *J* = 8.5, 9.0 Hz, H-3′), [4.34 (1H, dd, *J* = 5.0, 12.0 Hz), 4.48 (1H, dd, *J* = 2.0, 12.0 Hz), H_2_-6′], 4.55 (1H, q like, ca. *J* = 7 Hz, H-16), 4.91 (1H, d, *J* = 8.0 Hz, H-1′), 5.29 (1H, d, *J* = 7.5 Hz, H-1″); ^13^C-NMR (C_5_D_5_N, 125 MHz) spectroscopy data: see Table 1. HRESI-TOF-MS: Positive-ion mode *m*/*z* 725.4117 [M + H]^+^ (calcd. for C_38_H_61_O_13_, 725.4107).

*(25S)-Yucca spirostanoside E_2_* (**2b**): White powder; [α]${}_{D}^{25}$ − 0.45° (*c =* 0.45, MeOH); IR ν_max_ (KBr) cm^−1^: 3398, 2930, 2869, 1707, 1454, 1375, 1246, 1162, 1074, 1041, 986, 919, 896. ^1^H-NMR (C_5_D_5_N, 500 MHz): δ 0.87 (3H, s, H_3_-19), [0.97 (1H, m), 1.35 (1H, m, overlapped), H_2_-7], 1.08 (3H, d, *J* = 8.0 Hz, H_3_-27), 1.09 (3H, s, H_3_-18), 1.16, 1.80 (1H each, both m, H_2_-6), 1.28, 1.71 (1H each, both m, H_2_-1), 1.34, 2.13 (1H each, both m, overlapped, H_2_-24), 1.38 (3H, d, *J* = 7.0 Hz, H_3_-21), 1.40, 1.85 (1H each, both m, overlapped, H_2_-2), 1.41, 1.83 (1H each, both m, overlapped, H_2_-23), 1.46 (1H, m, H-14), 1.59 (1H, m, H-25), 1.61, 2.13 (1H each, both m, H_2_-15), 1.74 (2H, m, overlapped, H_2_-4), 1.75 (1H, m, overlapped, H-9), 1.83 (1H, m, overlapped, H-8), 1.89 (1H, m, H-20), 2.09 (1H, m, H-5), [2.20 (1H, dd, *J* = 5.0, 14.5 Hz), 2.37 (1H, dd, *J* = 14.5, 14.5 Hz), H_2_-11], 2.80 (1H, dd, *J* = 7.0, 8.5 Hz, H-17), [3.38 (1H, br. d, ca. *J* = 12 Hz), 4.05 (1H, dd, *J* = 3.5, 11.5 Hz), H_2_-26], [3.70 (1H, dd, *J* = 11.0, 11.0 Hz), 4.31 (1H, m, overlapped), H_2_-5″], 3.91 (1H, m, H-5′), 4.04 (1H, dd, *J* = 7.5, 8.0 Hz, H-2″), 4.08 (1H, dd, *J* = 7.5, 8.5 Hz, H-2′), 4.15 (1H, dd, *J* = 8.0, 9.0 Hz, H-3″), 4.17 (1H, m, H-4″), 4.19 (1H, dd, *J* = 9.0, 9.0 Hz, H-4′), 4.26 (1H, dd, *J* = 8.5, 9.0 Hz, H-3′), 4.30 (1H, m, overlapped, H-3), [4.34 (1H, dd, *J* = 5.0, 11.5 Hz), 4.49 (1H, dd, *J* = 2.0, 11.5 Hz), H_2_-6′], 4.52 (1H, m, H-16), 4.92 (1H, d, *J* = 7.5 Hz, H-1′), 5.31 (1H, d, *J* = 7.5 Hz, H-1″); ^13^C-NMR (C_5_D_5_N, 125 MHz) spectroscopy data: see Table 1. HRESI-TOF-MS: Positive-ion mode *m*/*z* 725.4117 [M + H]^+^ (calcd. for C_38_H_61_O_13_, 725.4107).

*(25R)-Yucca spirostanoside E_3_* (**3a**): White powder; [α]${}_{D}^{25}$ − 12.0° (*c =* 0.50, MeOH); IR ν_max_ (KBr) cm^−1^: 3426, 2927, 2870, 1707, 1456, 1376, 1238, 1161, 1072, 1043, 981, 920, 898, 865. ^1^H-NMR (C_5_D_5_N, 500 MHz): δ 0.70 (3H, d, *J* = 6.0 Hz, H_3_-27), 0.93, 1.31 (1H each, both m, H_2_-7), 1.01 (3H, s, H_3_-19), 1.09 (3H, s, H_3_-18), [1.21 (1H, m), 1.84 (1H each, both m), H_2_-6], [1.28 (1H, m), 1.72 (1H, m, overlapped), H_2_-1], 1.37 (3H, d, *J* = 7.0 Hz, H_3_-21), 1.37, 1.82 (1H each, both m, overlapped, H_2_-2), 1.44 (1H, m, H-14), 1.58 (2H, m, overlapped, H_2_-24), 1.58 (1H, m, overlapped, H-25), [1.64 (1H, m), 1.72 (1H, m, overlapped), H_2_-23], [1.64 (1H, m), 2.12 (1H, ddd, *J* = 6.0, 7.0, 12.5 Hz), H_2_-15], 1.73 (2H, m, overlapped, H_2_-4), 1.74 (1H, m, H-9), 1.84 (1H, m, overlapped, H-8), 1.94 (1H, quin, *J* = 7.0 Hz, H-20), [2.20 (1H, dd, *J* = 4.5, 14.0 Hz), 2.38 (1H, dd, *J* = 14.0, 14.0 Hz), H_2_-11], 2.25 (1H, m, H-5), 2.82 (1H, dd, *J* = 6.5, 8.5 Hz, H-17), [3.50 (1H, dd, *J* = 10.5, 10.5 Hz), 3.59 (1H, dd, *J* = 4.0, 10.5 Hz), H_2_-26], 3.86 (1H, m, H-5′), 3.98 (1H, m, H-5″), 4.09 (1H, dd, *J* = 8.0, 9.0 Hz, H-2″), 4.18 (1H, dd, *J* = 9.0, 9.0 Hz, H-4′), 4.23 (1H, dd, *J* = 7.5, 8.5 Hz, H-2′), 4.26 (1H, dd, *J* = 9.0, 9.0 Hz, H-3″), 4.28 (1H, m, H-3), 4.32 (1H, m, overlapped, H-3′), 4.32 (1H, m, overlapped, H-4″), [4.34 (1H, dd, *J* = 4.5, 11.5 Hz), 4.51 (1H, m, overlapped), H_2_-6′], [4.50 (1H, m, overlapped), 4.57 (1H, dd, *J* = 2.0, 12.0 Hz), H_2_-6″], 4.55 (1H, m, H-16), 4.93 (1H, d, *J* = 7.5 Hz, H-1′), 5.40 (1H, d, *J* = 8.0 Hz, H-1″); ^13^C-NMR (C_5_D_5_N, 125 MHz) spectroscopy data: see Table 1. HRESI-TOF-MS: Positive-ion mode *m*/*z* 777.4037 [M + Na]^+^ (calcd. for C_39_H_62_O_14_Na, 777.4032).

*(25S)-Yucca spirostanoside E_3_* (**3b**): White powder; [α]${}_{D}^{25}$ − 8.2° (*c =* 2.7, MeOH); IR ν_max_ (KBr) cm^−1^: 3408, 2929, 2987, 1704, 1451, 1373, 1171, 1075, 1032, 990, 919, 896, 850. ^1^H-NMR (C_5_D_5_N, 500 MHz): δ 0.92 (3H, s, H_3_-19), [0.94 (1H, m), 1.31 (1H, m, overlapped), H_2_-7], 1.07 (3H, d, *J* = 7.5 Hz, H_3_-27), 1.08 (3H, s, H_3_-18), 1.22, 1.84 (1H each, both m, H_2_-6), 1.29, 1.72 (1H each, both m, H_2_-1), 1.31, 1.86 (1H each, both m, overlapped, H_2_-2), 1.37 (3H, d, *J* = 7.0 Hz, H_3_-21), 1.38, 2.13 (1H each, both m, overlapped, H_2_-24), 1.43 (1H, m, H-14), 1.43, 1.91 (1H each, both m, H_2_-23), [1.59 (1H, m, overlapped), 2.10 (1H, m), H_2_-15], 1.60 (1H, m, overlapped, H-25), 1.74 (1H, m, overlapped, H-9), 1.83 (2H, m, overlapped, H_2_-4), 1.84 (1H, m, overlapped, H-8), 1.88 (1H, m, overlapped, H-20), 2.26 (1H, m, H-5), [2.19 (1H, dd, *J* = 5.0, 14.0 Hz), 2.37 (1H, dd, *J* = 14.0, 14.0 Hz), H_2_-11], 2.79 (1H, dd, *J* = 6.5, 8.5 Hz, H-17), [3.37 (1H, br. d, ca. *J* = 12 Hz), 4.06 (1H, dd, *J* = 2.5, 11.5 Hz), H_2_-26], 3.87 (1H, m, H-5′), 3.97 (1H, m, H-5″), 4.09 (1H, dd, *J* = 8.0, 8.0 Hz, H-2″), 4.18 (1H, dd, *J* = 9.0, 9.0 Hz, H-4′), 4.23 (1H, dd, *J* = 7.5, 8.5 Hz, H-2′), 4.24 (1H, m, H-3), 4.26 (1H, dd, *J* = 8.0, 9.0 Hz, H-3″), 4.31 (1H, m, overlapped, H-3′), 4.31 (1H, m, overlapped, H-4″), [4.34 (1H, dd, *J* = 5.0, 11.5 Hz), 4.50 (1H, m, overlapped), H_2_-6′], [4.49 (1H, m, overlapped), 4.56 (1H, dd, *J* = 3.0, 12.0 Hz), H_2_-6″], 4.51 (1H, m, H-16), 4.93 (1H, d, *J* = 7.5 Hz, H-1′), 5.40 (1H, d, *J* = 8.0 Hz, H-1″); ^13^C-NMR (C_5_D_5_N, 125 MHz) spectroscopy data: see Table 1. HRESI-TOF-MS: Positive-ion mode *m*/*z* 777.4035 [M + Na]^+^ (calcd. for C_39_H_62_O_14_Na, 777.4035).

### 3.4. Acid Hydrolysis of ***1a***–***3a***, ***1b***–***3b***

The solution of **1a**–**3a**, **1b**–**3b** (each 2.0 mg) in 1 M HCl (1.0 mL) was treated by using the same method as described in reference [5]: They were heated under reflux for 3 h. Then each reaction mixture was detected by CH_3_CN–H_2_O (75:25, *v*/*v*; flow rate 1.0 mL/min). As a result, d-glucose was found from the aqueous phase of **1a**–**3a**, **1b**–**3b**, and d-xylose was detected from **2a** and **3b** by comparison of their *t*_R_ and optical rotation with those of the authentic sample, d-glucose (*t*_R_ 12.4 min (positive)), d-xylose (*t*_R_ 6.1 min (positive)).

### 3.5. Bioassay

#### 3.5.1. Materials

SW620 cell line was obtained from Cell Resource Center of Institute of Basic Medical Sciences, Chinese Academy of Medical Sciences & Peking Union Medical College (Beijing, China). Fetal Bovine Serum (FBS) was purchased from Biological Industries (Beit-Haemek, Israel). Roswell Park Memorial Institute (RPMI) 1640 medium was from Corning (Shanghai, China). Penicillin, streptomycin, and methyl thiazolyl tetrazolium (MTT) were ordered from Thermo Fisher Scientific (Shanghai, China). Dimethyl sulfoxide (DMSO) and 5-fluorouracil were purchased from Sigma-Aldrich (St. Louis, MO, USA).

#### 3.5.2. MTT Assay

The inhibitory effects of *Y. schidigera* 70% EtOH extract, *Y. schidigera* 95% EtOH eluate, *Y. schidigera* H_2_O eluate, as well as 25*R*/*S*-spirostanol saponin diastereomers **1a**–**6a**, **1b**–**6b** were tested for their individual inhibitory activities on the growth of SW620 cell lines. Cell viability in the presence or absence of tested samples (with the positive control, 5-fluorouracil) was determined using the MTT method [9].

The SW620 cell lines were maintained in RPMI 1640 medium supplemented with 10% FBS, 100 U/mL penicillin and 100 μg/mL streptomycin and kept in a humidified atmosphere of 95% air and 5% CO_2_ at 37 °C. For cell viability determination, exponentially growing cells were harvested and plated in 96-well plates (5 × 10^4^/mL) in RPMI 1640 medium for 24 h. After the cells had been washed with PBS, the medium was changed to serially diluted test samples in RPMI. After 48 h of incubation, the cells were washed twice with PBS, and MTT solution was added and incubated for 4 h at 37 °C. Then, MTT was removed. After 100 μL DMSO was added, the 96-well plate was shaken (90 *R*/*S*) for 5 min at room temperature under avoiding light, then the absorbance was determined at 490 nm by microplate reader. The tested compounds were independently performed four times. Values are expressed as mean ± S.D. The IC_50_ values were statistically determined using the SPSS 11.0 software (International business machines corporation (IBM Co.), Armonk, NY, USA).

## 4. Conclusions

Analysis of spirostanol saponins is usually performed by ultra-high performance supercritical fluid chromatography (UHPSFC) and ultra-high performance liquid chromatography (UHPLC) [10], which are not suitable for mass preparation. Until now, successful preparation examples for (25*R*/*S*)-spiromeric epimers were accomplished by SFC isolation [11,12] usually. Meanwhile, it is very rare still for preparing this type of *R*/*S* mixture using HPLC methods [13,14,15,16]. On the other hand, among the rare isolation examples, all of them are only for the isolation of C_12_ unsubstituted 25*R*/*S*-spirostanol saponin diastereomers, but no reference was found to separate C_12_ carbonylated 25*R*/*S*-ones. Though the emergence of SFC technology provides a new idea for the qualitative and quantitative analysis of drugs, there are still some difficulties for the further promotion and application of this technology [17]: (1) the types of stationary phases are rare, compounds with strong polarity show poor separation; (2) stationary phase, mobile phase and compounds often do not have good compatibility; (3) not all samples have good solubility in CO_2_; (4) when combined with mass spectrometry due to the delayed effect of the porous structure of the stationary phase, a large flow rate is often required, so desirable environmental protection objectives (less mobile phase usage) cannot be achieved. Therofore, it is very important to establish a simple, universal, fast and mass separation analysis method for 25*R*/*S*-spirostanol saponin diastereomers.

In this paper, the liquid chromatographic retention behaviors of C_12_ carbonylated and C_12_ unsubstituted 25*R*/*S*-spirostanol saponin diastereomers on three kinds of stationary phases (C_8_, C_18_, C_30_ columns) and with two kinds of mobile phases (MeOH-1% CH_3_COOH and CH_3_CN-1% CH_3_COOH) were investigated. A C_30_ column was firstly found to be more suitable for the separation of this kind of diastereomers than C_8_ and C_18_ column. Meanwhile, the analysis results indicated that both CH_3_CN-1% CH_3_COOH and MeOH-1% CH_3_COOH eluate systems were selective to C_12_ unsubstituted 25*R*/*S*-spirostanol saponin diastereomers, while MeOH-1% CH_3_COOH possessed better selectivity for C_12_ carbonylated ones. Compared with SFC, this method is more simple and versatile. Since 25*R*/*S*-spirostanol saponin diastereomers are widely distributed in natural herbs, this research provides a rapid and reliable method for the isolation of similar compounds from plant materials.

On the basis of the abovementioned analysis method, six pairs of 25*R*/*S*-spirostanol saponin diastereomers **1a**–**6a**, **1b**–**6b** were isolated from *Y. Schidigera* succefully. Among them, **1a**–**3a**, **1b**–**3b** were new compounds and **4a** and **4b** were isolated from *Y. schidigera* for the first time. The NMR spectrum of compounds: **1a**–**3a**, **1b**–**3b** are in the Supplementary Material.

On the other hand, the study of inhibitory activity and structure-activity relationships of 25*R*/*S*-spirostanol saponin diastereomers from *Y. schidigera* on the growth of SW620 cells will lay a foundation for the further mechanism studies, and it will supply an example for searching for anti-colon cancer drugs in natural products. What’s more, the summary of the structure-activity relationships affords a basis for structural modification, and semi- or total synthesis of new anti-cancer drugs.

## Figures and Tables

**Table 1:** ^13^C-NMR data for **1a**–**3a** and **1b**–**3b** in C_5_D_5_N.

No.	1a	1b	2a	2b	3a	3b	No.	1a	1b	2a	2b	3a	3b
1	30.6	30.6	30.6	30.6	30.6	30.6	21	14.0	13.8	14.0	13.8	13.9	13.8
2	26.7	26.7	26.6	26.7	26.6	26.6	22	109.3	109.8	109.3	109.8	109.3	109.8
3	73.9	73.9	74.0	74.0	74.9	74.9	23	31.8	26.4	31.8	26.4	31.9	26.4
4	30.2	30.2	30.1	30.1	30.7	30.7	24	29.2	26.1	29.2	26.2	29.3	26.2
5	36.5	36.5	36.5	36.5	36.4	36.4	25	30.5	27.5	30.5	27.5	30.6	27.5
6	26.8	26.8	26.8	26.8	26.8	26.8	26	66.9	65.2	66.9	65.2	67.0	65.2
7	26.4	26.4	26.4	26.4	26.4	26.4	27	17.3	16.3	17.3	16.3	17.3	16.3
8	34.7	34.7	34.7	34.7	34.7	34.7	1′	102.9	102.9	102.3	102.4	102.0	101.8
9	41.9	41.9	41.9	41.9	42.0	42.0	2′	75.3	75.3	74.2	74.3	83.1	83.1
10	35.7	35.7	35.7	35.7	35.8	35.8	3′	78.7	78.7	87.7	87.8	78.2	78.2
11	37.7	37.7	37.7	37.8	37.8	37.8	4′	71.7	71.7	69.5	69.5	71.6	71.6
12	213.0	213.0	213.0	213.0	213.0	213.0	5′	78.4	78.4	78.1	78.2	78.3	78.3
13	55.6	55.6	55.6	55.6	55.7	55.6	6′	62.8	62.8	62.3	62.4	62.7	62.7
14	56.0	56.0	56.0	56.0	56.1	56.1	1″			106.3	106.4	106.0	106.0
15	31.5	31.4	31.5	31.4	31.5	31.5	2″			75.3	75.4	77.1	77.1
16	79.8	79.9	79.8	79.9	79.8	79.9	3″			78.2	78.2	78.0	78.0
17	54.3	54.2	54.3	54.2	54.4	54.2	4″			70.9	70.9	71.9	71.9
18	16.1	16.1	16.1	16.1	16.1	16.1	5″			67.4	67.4	78.6	78.6
19	23.0	23.0	23.1	23.1	23.2	23.2	6″					63.0	63.0
20	42.6	43.1	42.6	43.1	42.7	43.2							

**Table 2:** The inhibitory effects of *Y. schidigera* extract, fractions, and 25*R*/*S*-spirostanol saponin diastereomers on the growth of SW620 cell.

Sample	IC_50_	Sample	IC_50_
Positive control	10.00 ± 0.15	**3a**	29.81 ± 0.21
*Y. schidigera* 70% EtOH extract	85.20 ± 0.95	**3b**	55.90 ± 0.78
*Y. schidigera* 95% EtOH eluate	93.04 ± 1.21	**4a**	>100
*Y. schidigera* H_2_O eluate	>100	**4b**	60.26 ± 4.53
**1a**	>100	**5a**	63.37 ± 0.70
**1b**	>100	**5b**	33.91 ± 1.27
**2a**	>100	**6a**	69.17 ± 1.24
**2b**	>100	**6b**	12.02 ± 1.43

n = 4; Positive control: 5-FU; IC_50_: μg/mL for *Y. schidigera* 70% EtOH extract, *Y. schidigera* 95% EtOH eluate, and *Y. schidigera* H_2_O eluate; μM for positive control and compounds**1a**–**6a** and **1b**–**6b**.

## Supplementary Materials

Supplementary materials are available online.
