# Supplementary material for: Separation and Bioactive Assay of 25R/S-Spirostanol Saponin Diastereomers from Yucca schidigera Roezl (Mojave) Stems
Source: Molecules. 2018 Oct 8;23(10):2562. doi: 10.3390/molecules23102562 (PMC6222657; doi:10.3390/molecules23102562)
Supplement: Supplementary file 1 [file molecules-23-02562-s001.pdf]

Separation and Bioactive Assay of 25*R*/*S*-Spirostanol Saponin Diastereomers from *Yucca schidigera* Roezl (Mojave) Stems

Lu Qu [1], Jingya Ruan [1], Song Wu [2], Peijian Huang [2], Jiejing Yan [1], Haiyang Yu [2], Yi Zhang [1,2,*], and Tao Wang [1,2,*]

[1] Tianjin State Key Laboratory of Modern Chinese Medicine, Tianjin University of Traditional Chinese Medicine, 312 Anshanxi Road, Nankai District,Tianjin 300193, China; qululuhan88@163.com (L.Q.); Ruanjy19930919@163.com (J.R.); 17320072093@163.com (J.Y.)

[2] Tianjin Key Laboratory of TCM Chemistry and Analysis, Institute of Traditional Chinese Medicine, Tianjin University of Traditional Chinese Medicine, 312 Anshanxi Road, Nankai District, Tianjin 300193, China; 1903533440@qq.com (S.W); hpjforever@sina.com (P.H.); hyyu@tjutcm.edu.cn (H.Y.)

* Correspondence: zhwwxzh@263.net (Y.Z.); wangtao@tjutcm.edu.cn (T.W.); Tel./Fax: +86-22-5959-6163 (Y.Z.); +86-22-59596168 (T.W.)

Figure S1 ^{1}H NMR (500MHz, C_5D_5N) spectrum of compound **1a** 3
Figure S2 ^{13}C NMR (125MHz, C_5D_5N) spectrum of compound **1a** 3
Figure S3 The DEPT 135 (C_5D_5N) spectrum of compound **1a** 4
Figure S4 ^{1}H ^{1}H COSY (C_5D_5N) spectrum of compound **1a** 4
Figure S5 The HSQC (C_5D_5N) spectrum of compound **1a** 5
Figure S6 The HMBC (C_5D_5N) spectrum of compound **1a** 5
Figure S7 The HRESI-TOF-MS spectrum of compound **1a** 6
Figure S8 ^{1}H NMR (500MHz, C_5D_5N) spectrum of compound **1b** 7
Figure S9 ^{13}C NMR (125MHz, C_5D_5N) spectrum of compound **1b** 7
Figure S10 The DEPT 135 (C_5D_5N) spectrum of compound **1b** 8
Figure S11 ^{1}H ^{1}H COSY (C_5D_5N) spectrum of compound **1b** 8
Figure S12 The HSQC (C_5D_5N) spectrum of compound **1b** 9
Figure S13 The HMBC (C_5D_5N) spectrum of compound **1b** 9
Figure S14 The HRESI-TOF-MS spectrum of compound **1b** 10
Figure S15 ^{1}H NMR (500MHz, C_5D_5N) spectrum of compound **2a** 11
Figure S16 ^{13}C NMR (125MHz, C_5D_5N) spectrum of compound **2a** 11
Figure S17 The DEPT 135 (C_5D_5N) spectrum of compound **2a** 12
Figure S18 ^{1}H ^{1}H COSY (C_5D_5N) spectrum of compound **2a** 12
Figure S19 The HSQC (C_5D_5N) spectrum of compound **2a** 13
Figure S20 The HMBC (C_5D_5N) spectrum of compound **2a** 13
Figure S21 The HRESI-TOF-MS spectrum of compound **2a** 14
Figure S22 ^{1}H NMR (500MHz, C_5D_5N) spectrum of compound **2b** 15
Figure S23 ^{13}C NMR (125MHz, C_5D_5N) spectrum of compound **2b** 15
Figure S24 The DEPT 135 (C_5D_5N) spectrum of compound **2b** 16
Figure S25 ^{1}H ^{1}H COSY (C_5D_5N) spectrum of compound **2b** 16
Figure S26 The HSQC (C_5D_5N) spectrum of compound **2b** 17
Figure S27 The HMBC (C_5D_5N) spectrum of compound **2b** 17
Figure S28 The HRESI-TOF-MS spectrum of compound **2b** 18
Figure S29 ^{1}H NMR (500MHz, C_5D_5N) spectrum of compound **3a** 19
Figure S30 ^{13}C NMR (125MHz, C_5D_5N) spectrum of compound **3a** 19
Figure S31 ^{1}H ^{1}H COSY (C_5D_5N) spectrum of compound **3a** 20
Figure S32 The HSQC (C_5D_5N) spectrum of compound **3a** 20
Figure S33 The HMBC (C_5D_5N) spectrum of compound **3a** 21
Figure S34 The HRESI-TOF-MS spectrum of compound **3a** 21
Figure S35 ^{1}H NMR (500MHz, C_5D_5N) spectrum of compound **3b** 22
Figure S36 ^{13}C NMR (125MHz, C_5D_5N) spectrum of compound **3b** 22
Figure S37 ^{1}H ^{1}H COSY (C_5D_5N) spectrum of compound **3b** 23
Figure S38 The HSQC (C_5D_5N) spectrum of compound **3b** 23
Figure S39 The HMBC (C_5D_5N) spectrum of compound **3b** 24
Figure S40 The HRESI-TOF-MS spectrum of compound **3b** 24

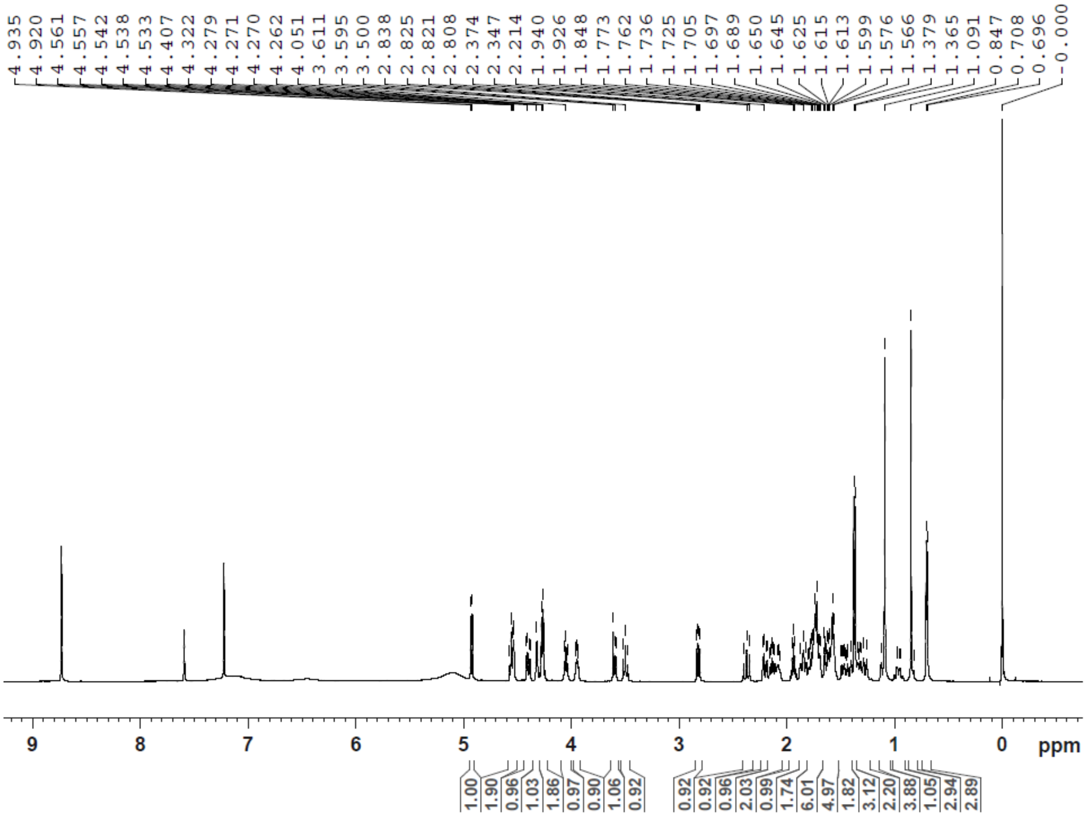

Figure S1 ^{1}H NMR (500MHz, C_5D_5N) spectrum of compound **1a**

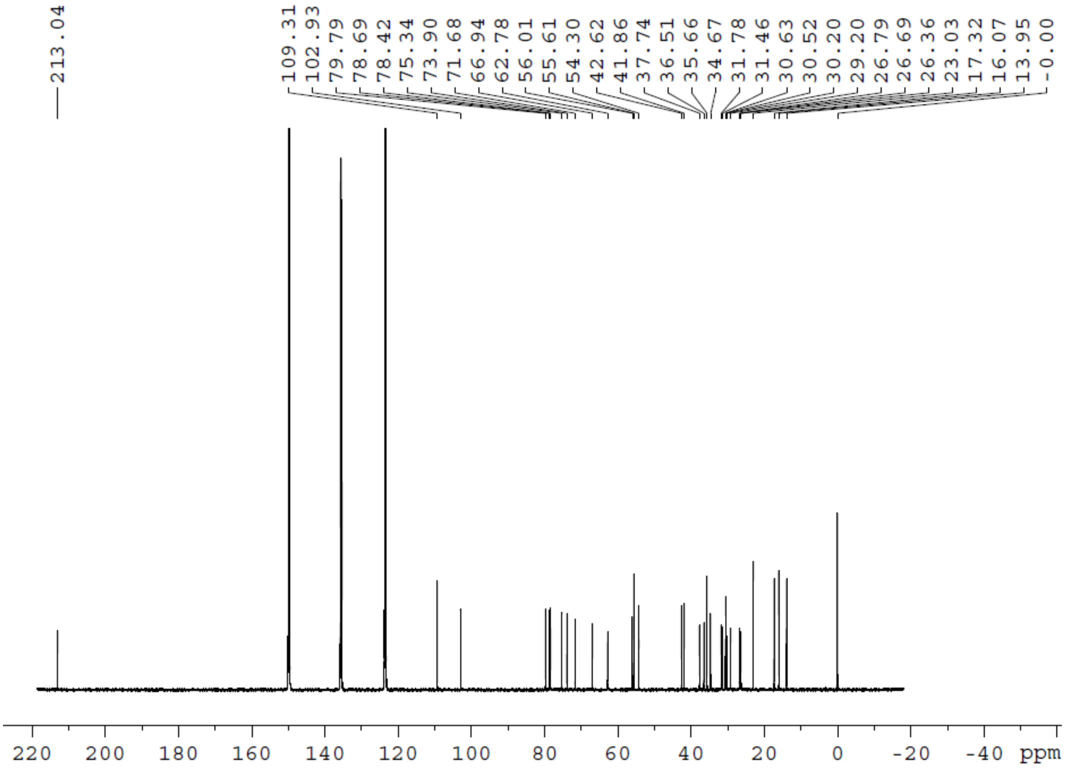

Figure S2 ^{13}C NMR (125MHz, C_5D_5N) spectrum of compound **1a**

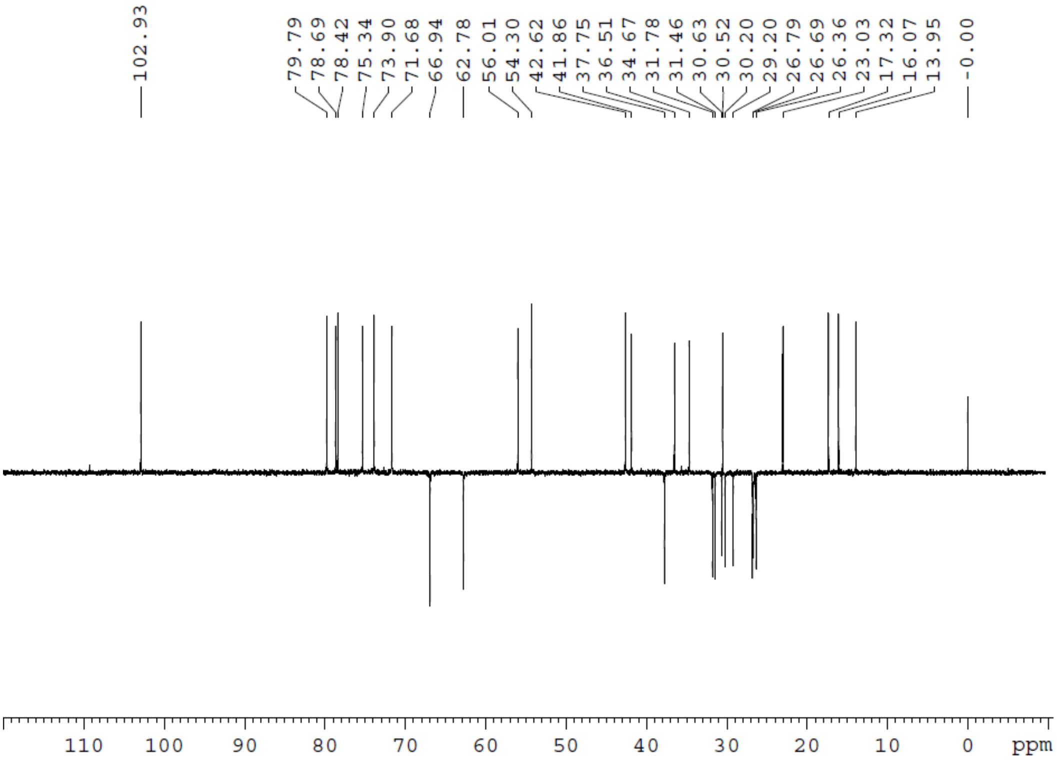

Figure S3 The DEPT 135 (C_5D_5N) spectrum of compound **1a**

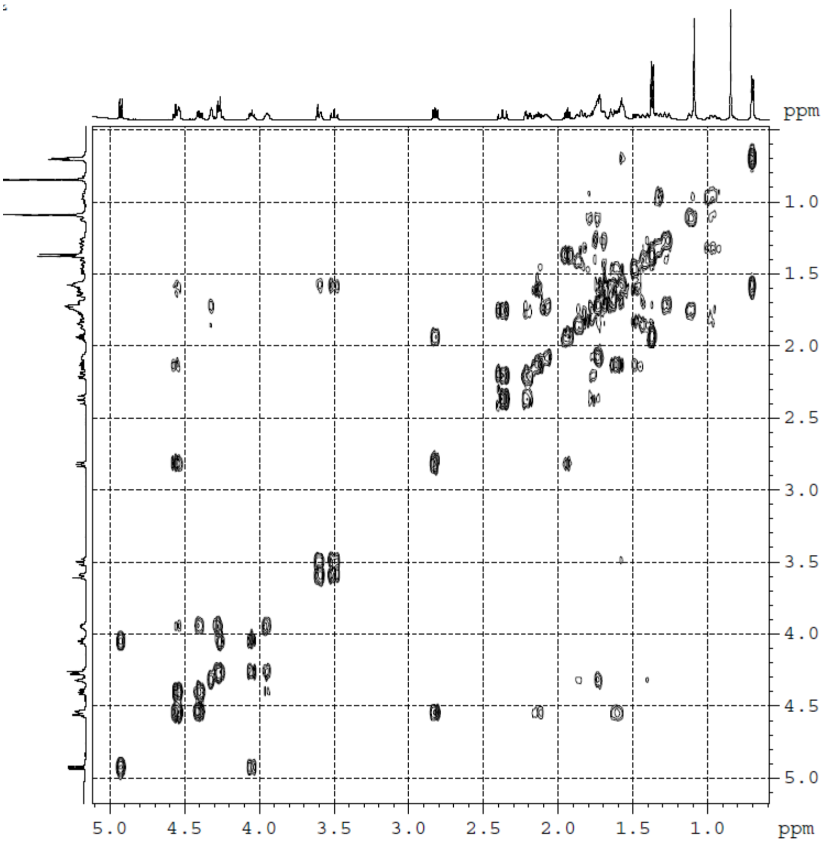

Figure S4 ^1H ^1H COSY (C_5D_5N) spectrum of compound **1a**

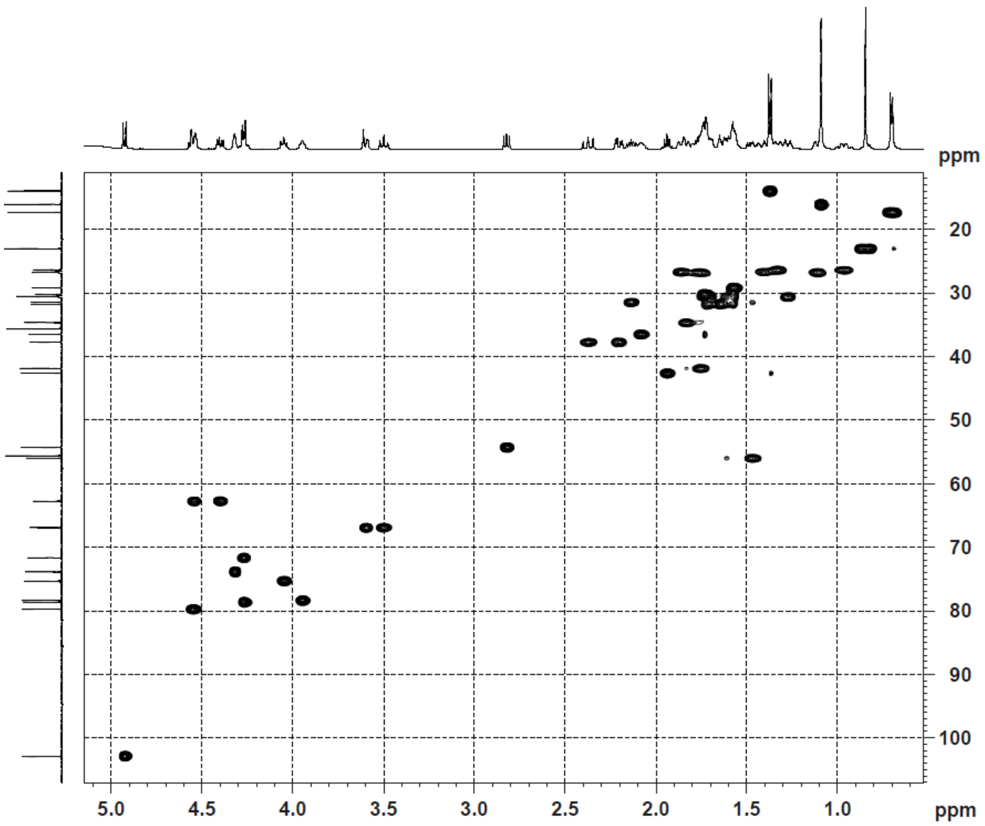

Figure S5 The HSQC (C_5D_5N) spectrum of compound **1a**

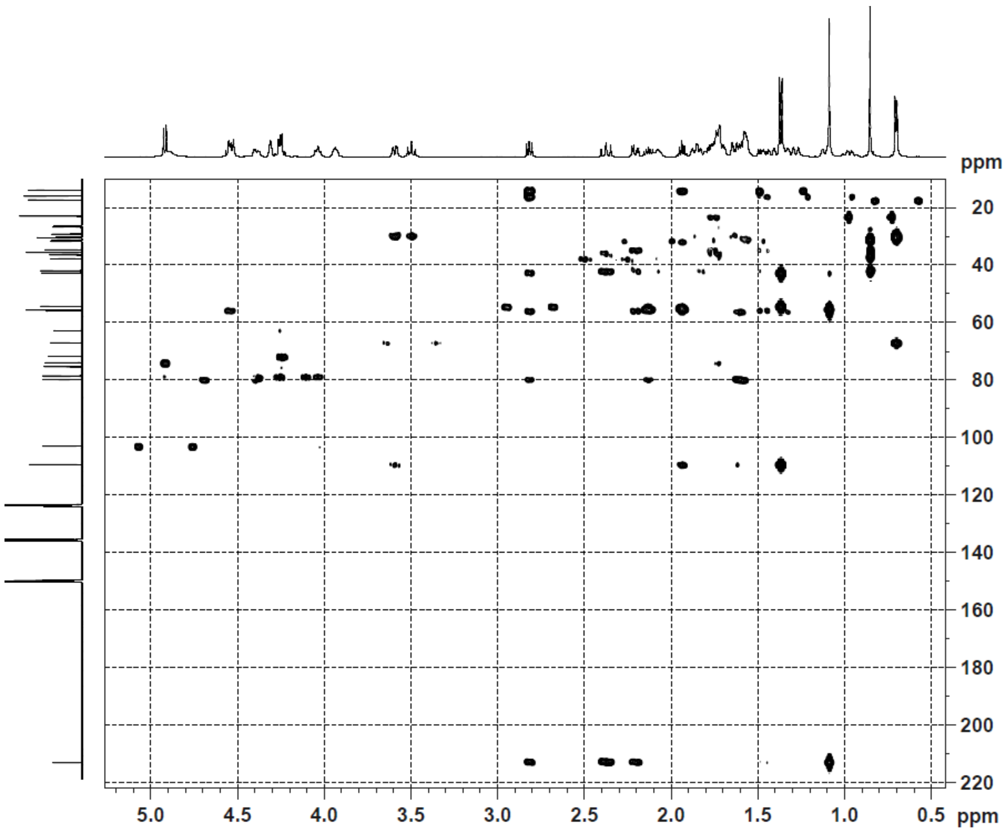

Figure S6 The HMBC (C_5D_5N) spectrum of compound **1a**

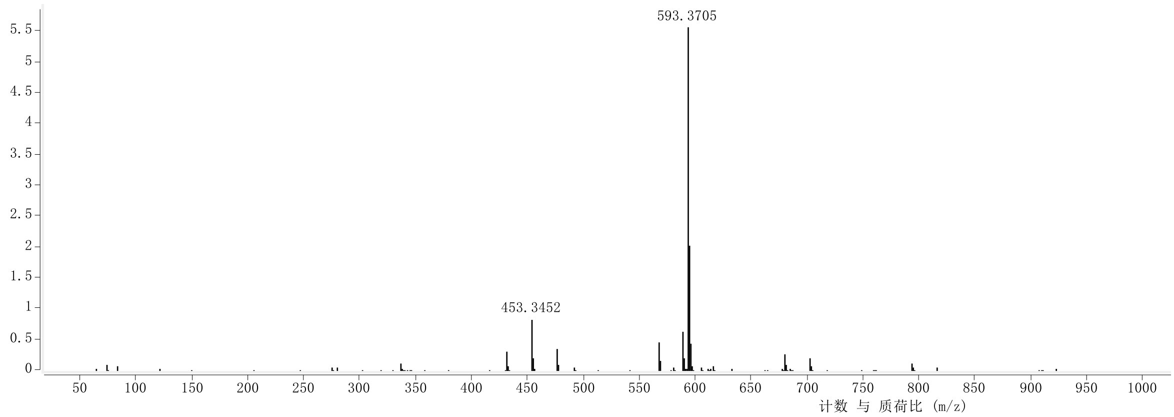

Figure S7 The HRESI-TOF-MS spectrum of compound **1a**

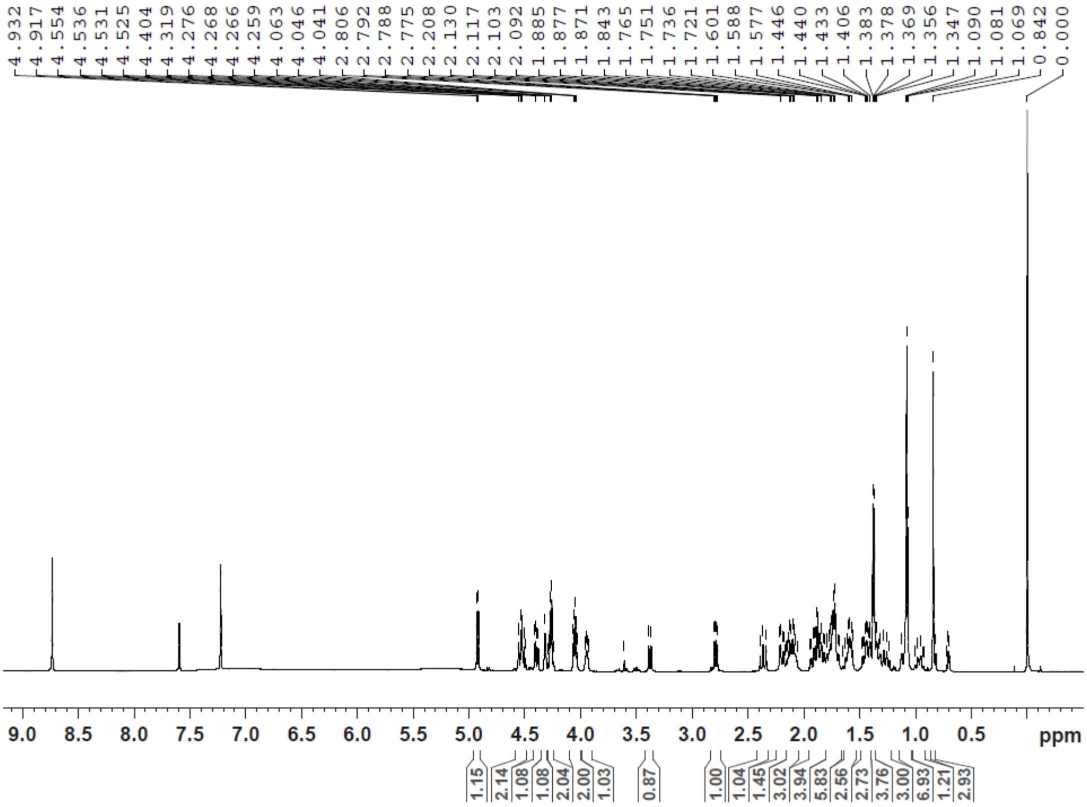

Figure S8 ^{1}H NMR (500MHz, C_5D_5N) spectrum of compound **1b**

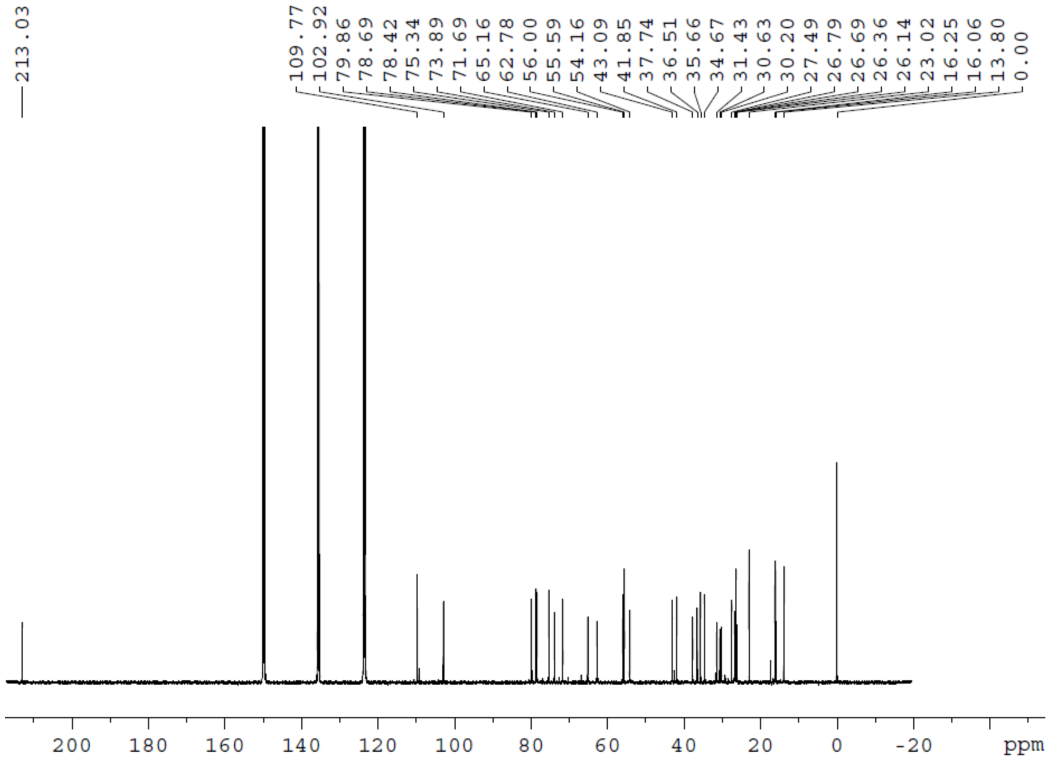

Figure S9 ^{13}C NMR (125MHz, C_5D_5N) spectrum of compound **1b**

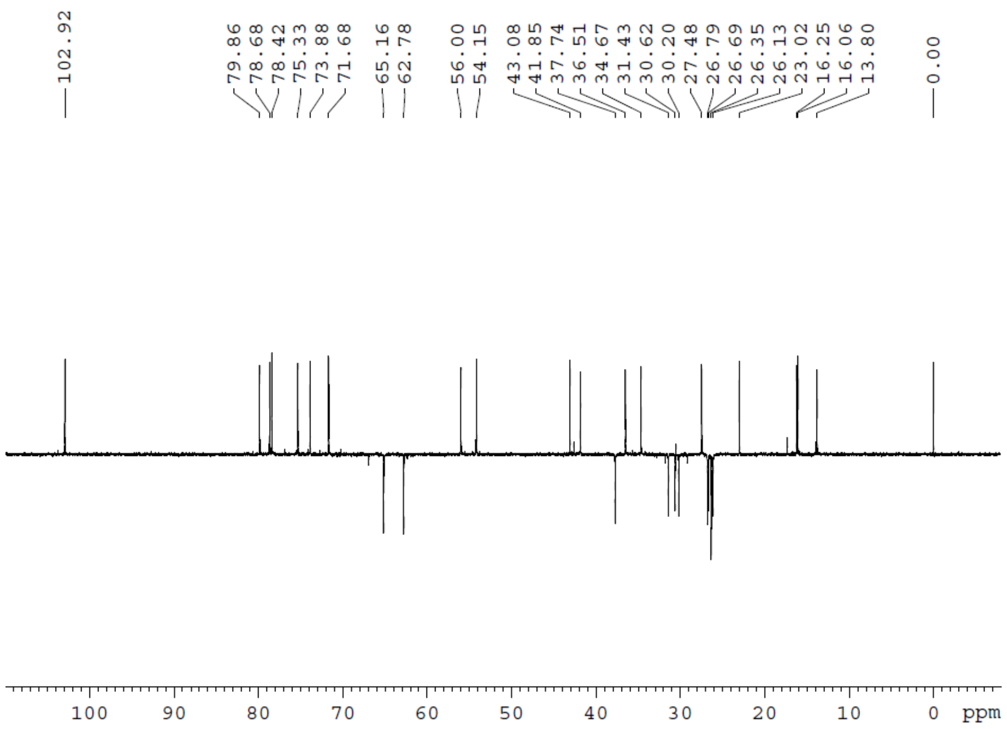

Figure S10 The DEPT 135 (C_5D_5N) spectrum of compound **1b**

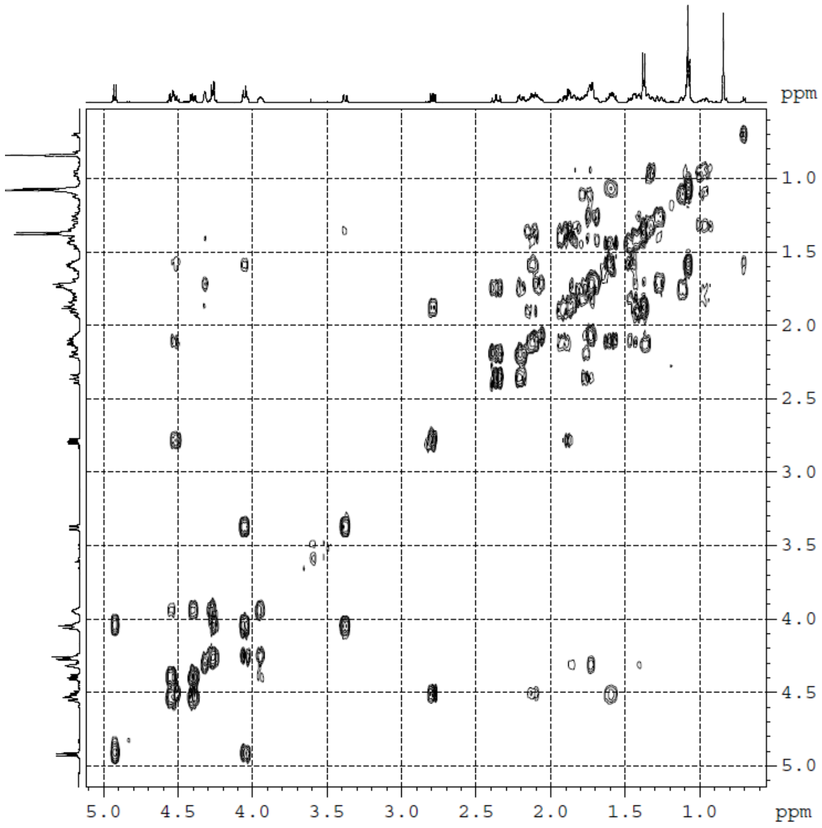

Figure S11 The ^{1}H ^{1}H COSY (C_5D_5N) spectrum of compound **1b**

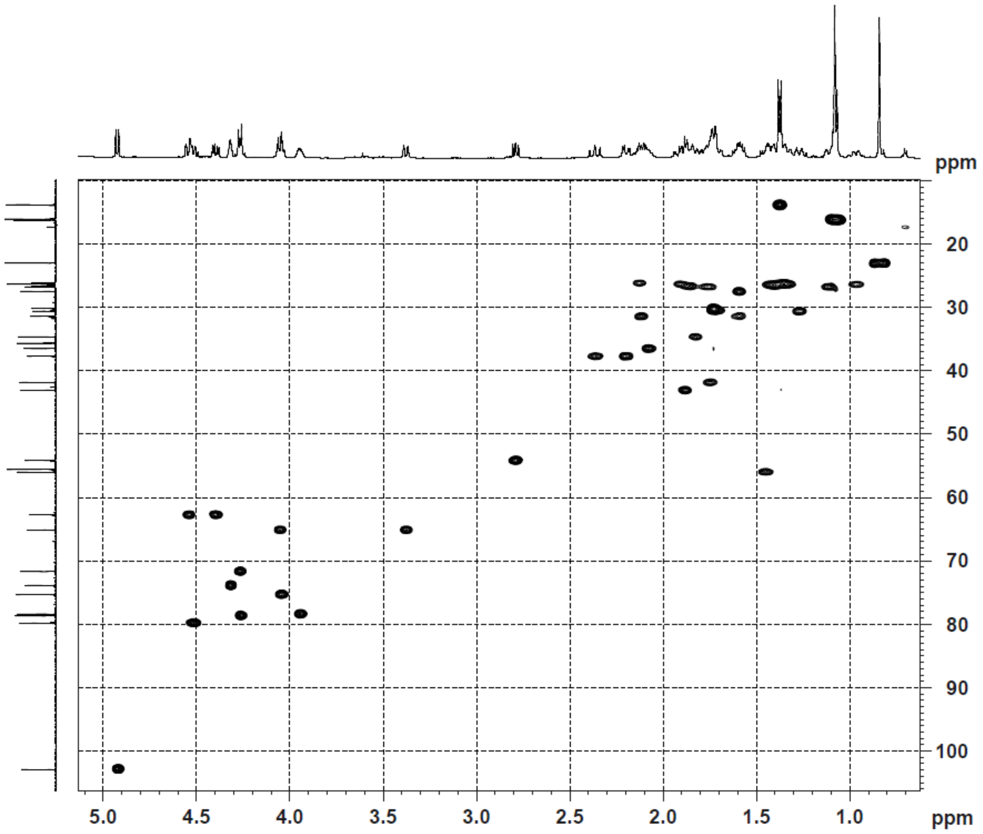

Figure S12 The HSQC (C_5D_5N) spectrum of compound **1b**

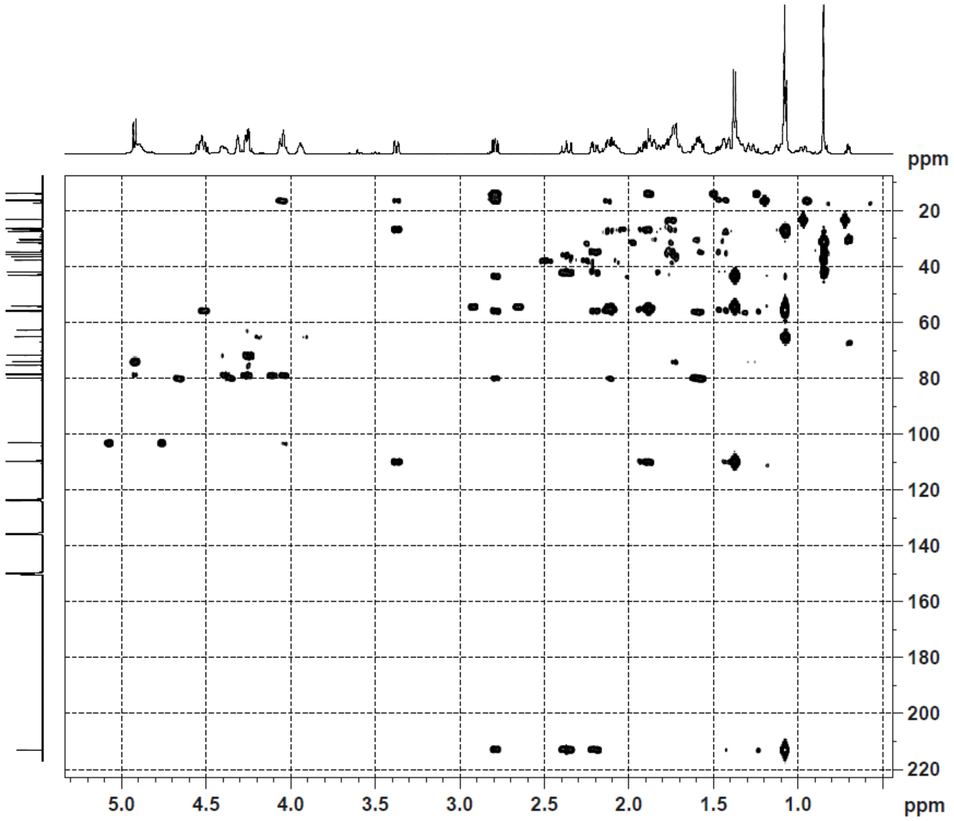

Figure S13 The HMBC (C_5D_5N) spectrum of compound **1b**

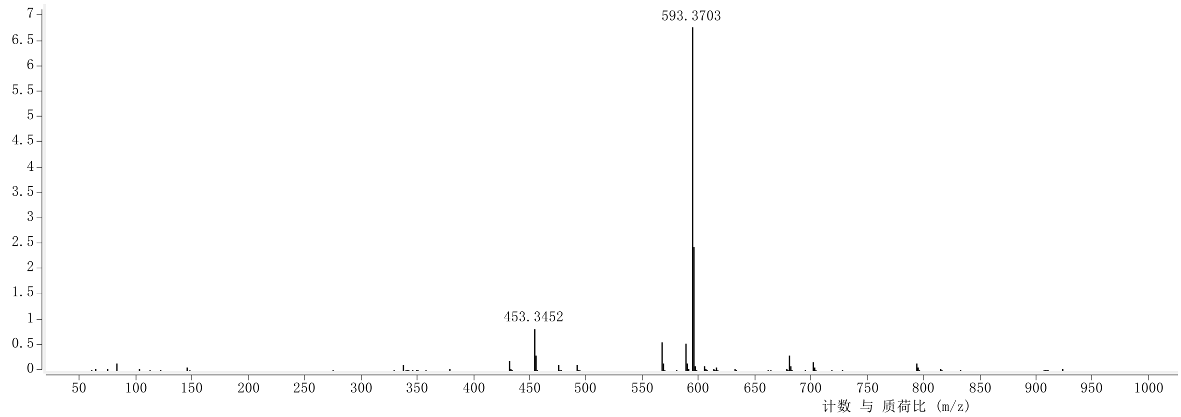

Figure S14 The HRESI-TOF-MS spectrum of compound **1b**

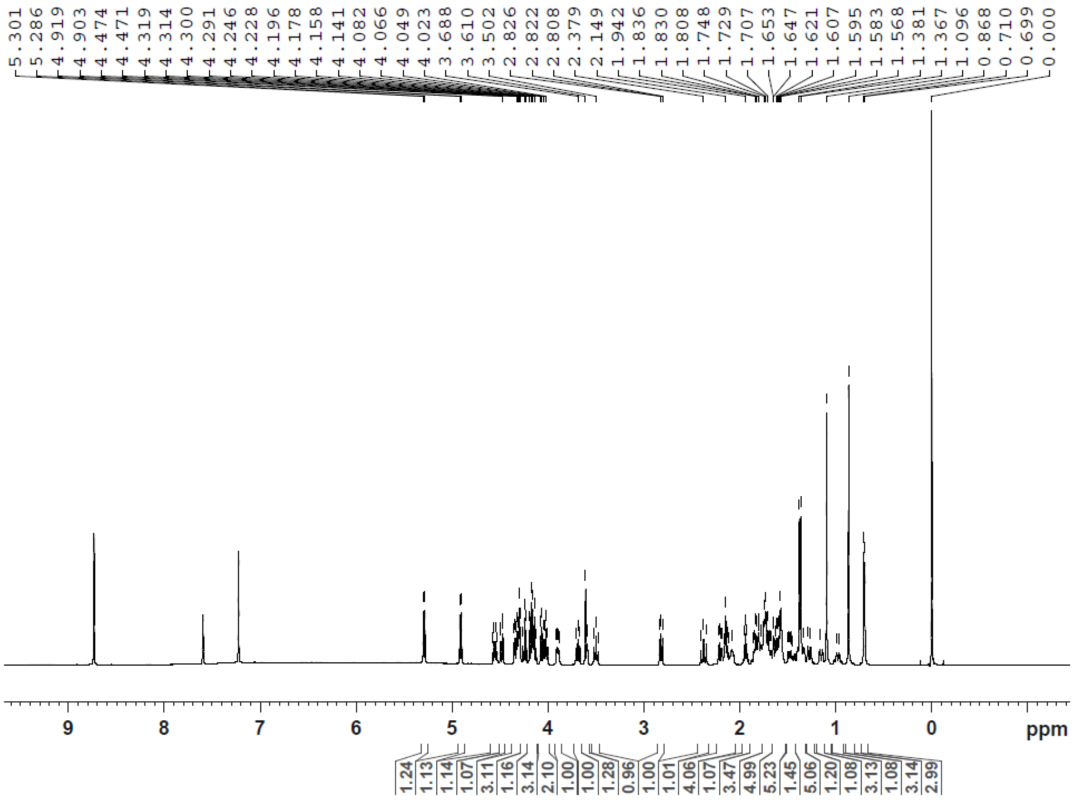

Figure S15 ^{1}H NMR (500MHz, C_5D_5N) spectrum of compound **2a**

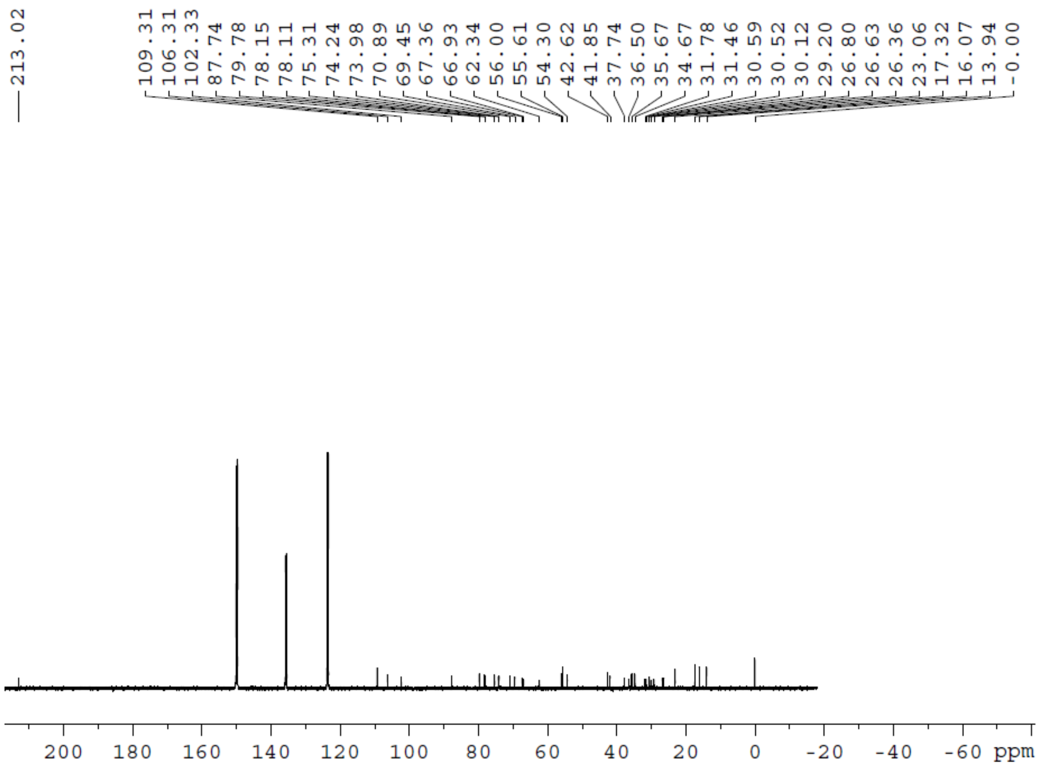

Figure S16 ^{13}C NMR (125MHz, C_5D_5N) spectrum of compound **2a**

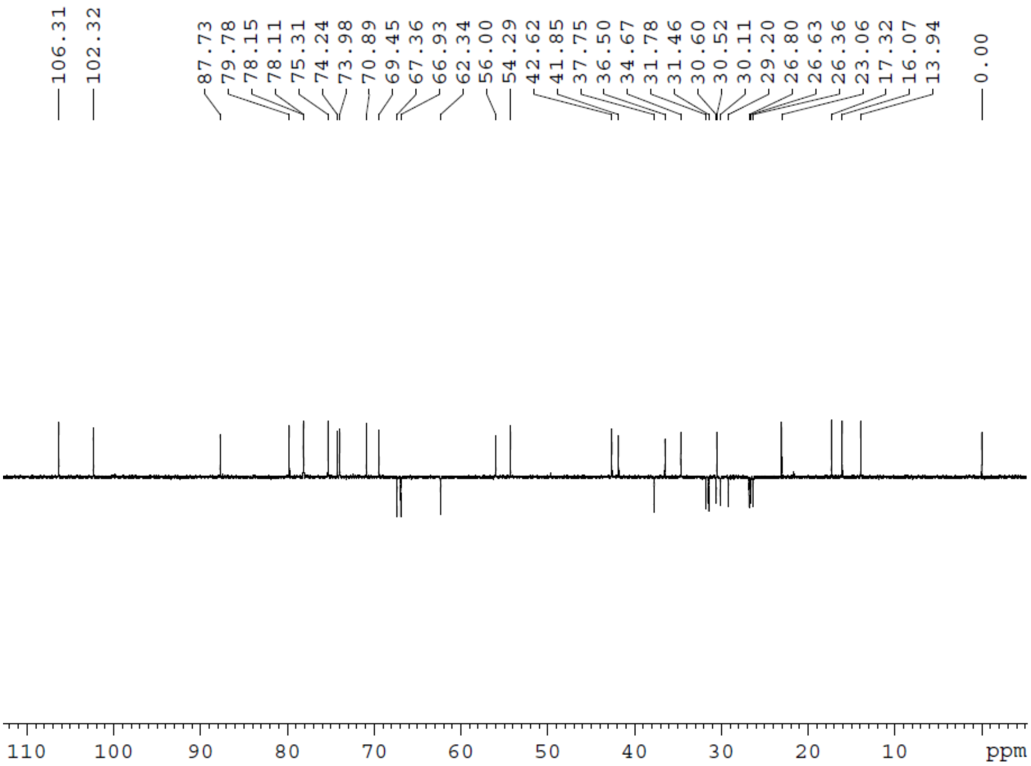

Figure S17 The DEPT 135 (C_5D_5N) spectrum of compound **2a**

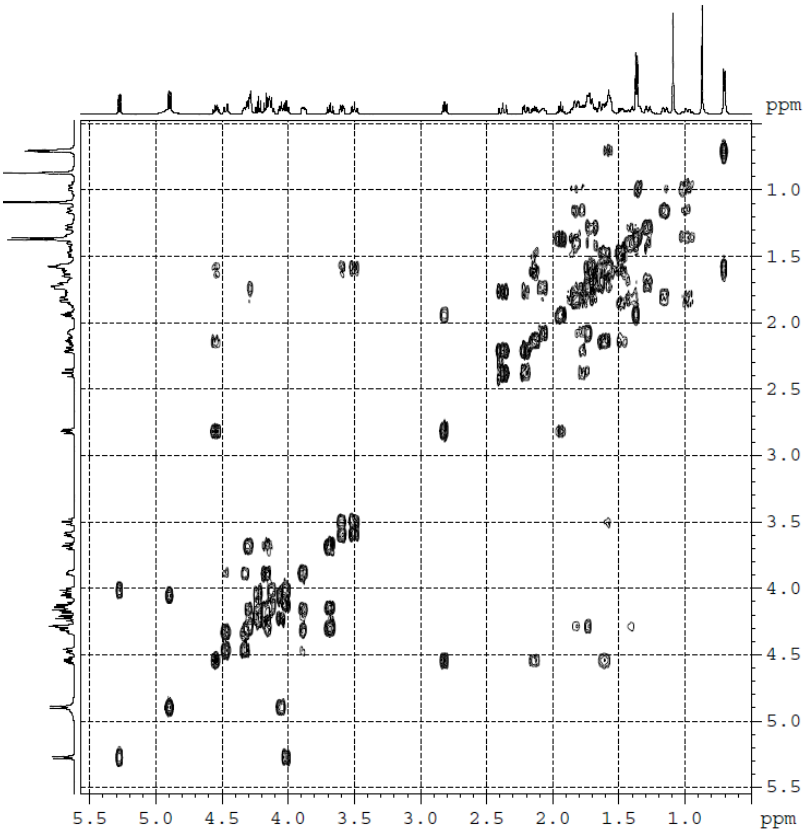

Figure S18 The ^1H ^1H COSY (C_5D_5N) spectrum of compound **2a**

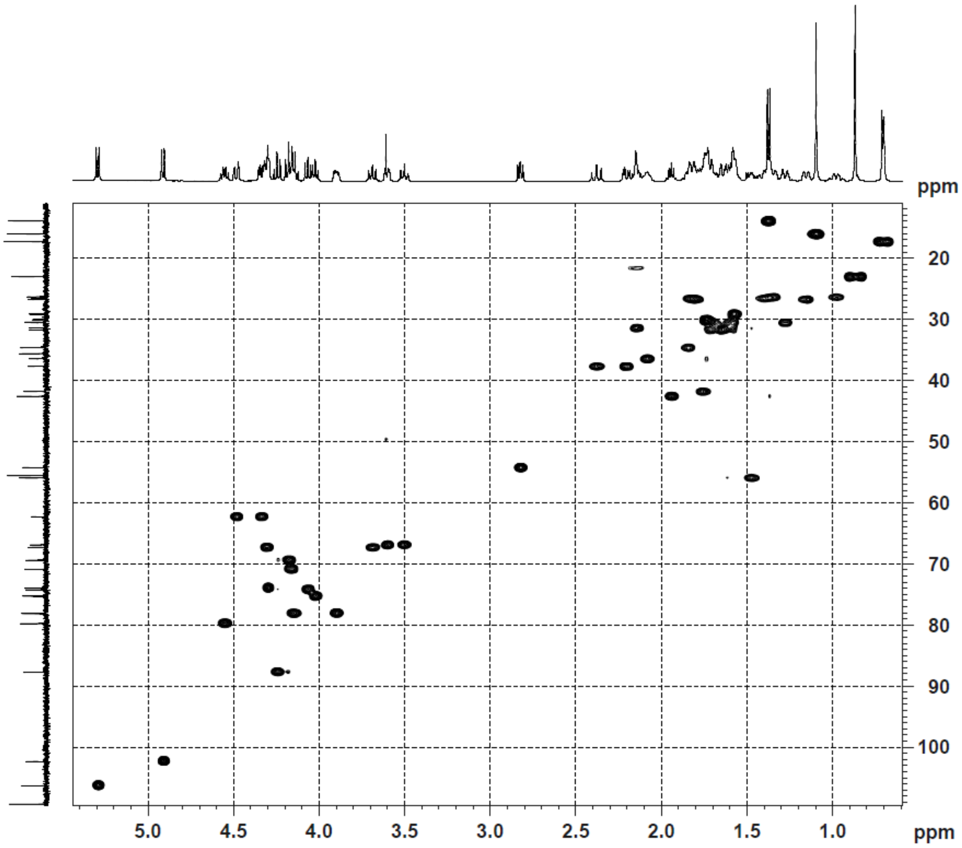

Figure S19 The HSQC (C_5D_5N) spectrum of compound **2a**

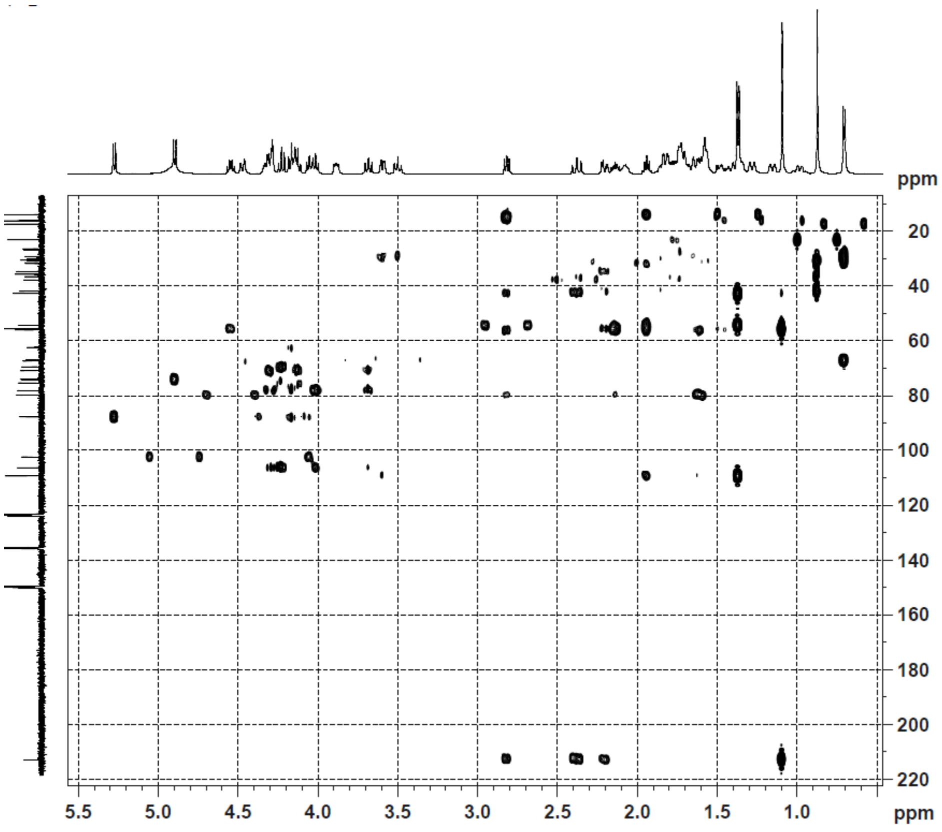

Figure S20 The HMBC (C_5D_5N) spectrum of compound **2a**

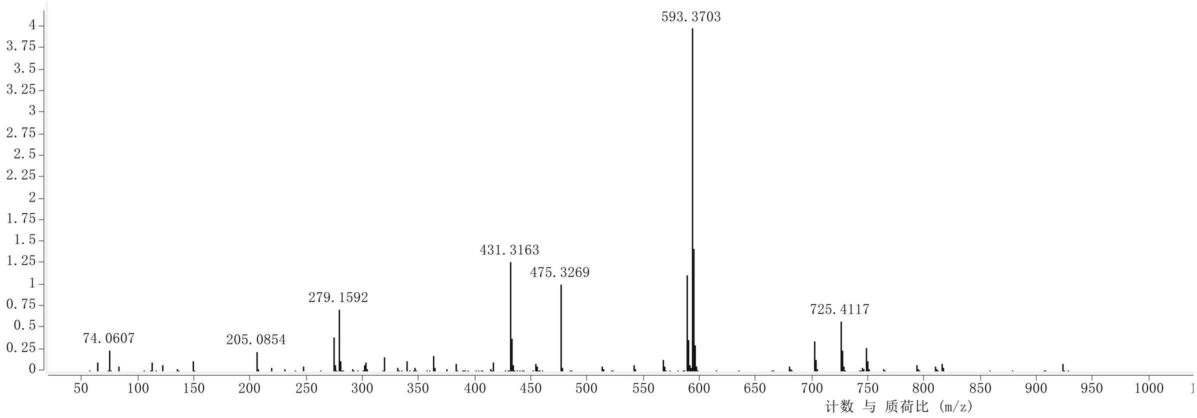

Figure S21 The HRESI-TOF-MS spectrum of compound **2a**

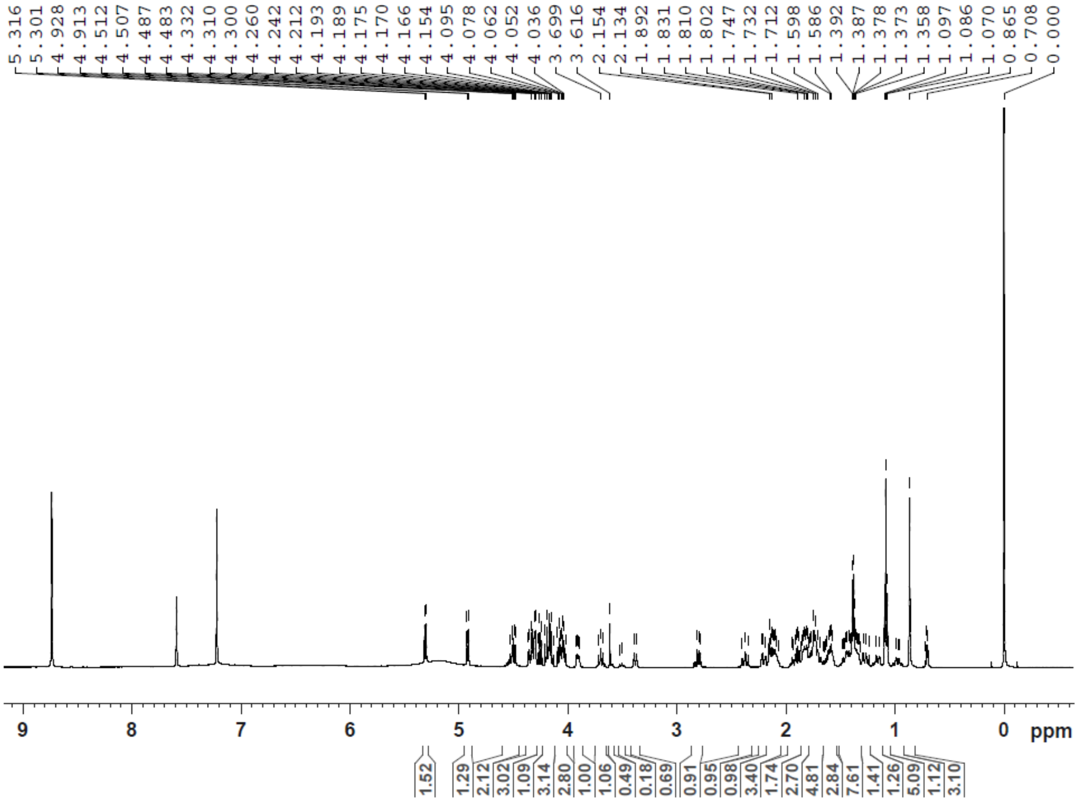

Figure S22 ^{1}H NMR (500MHz, C_5D_5N) spectrum of compound **2b**

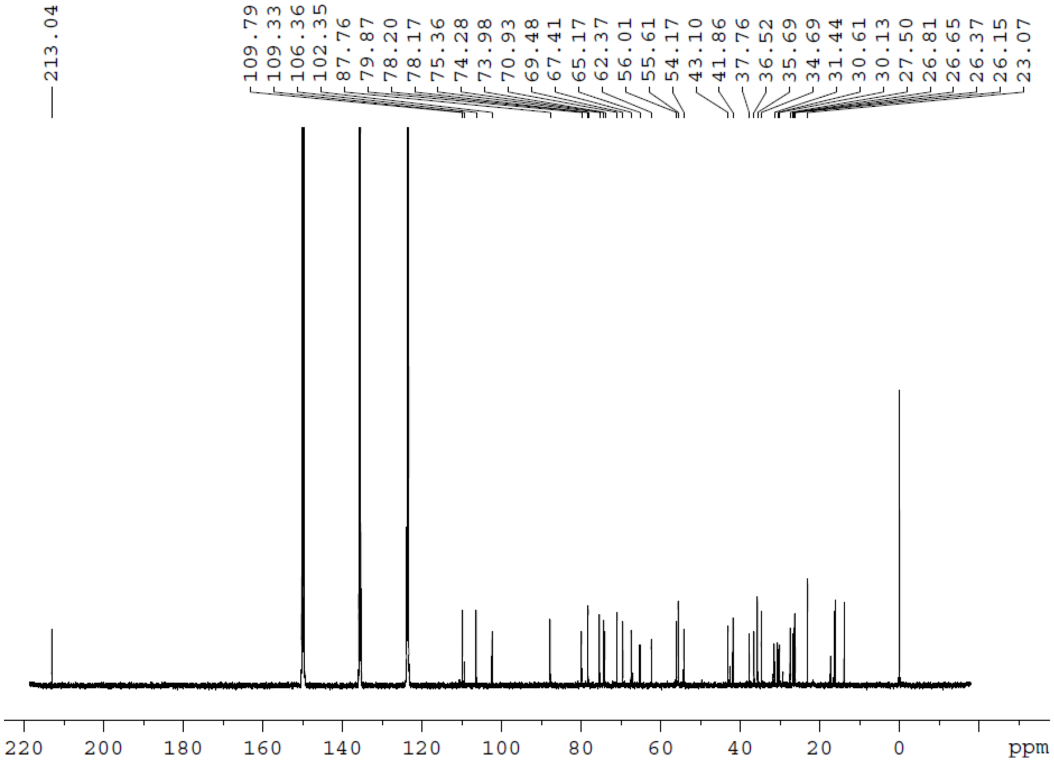

Figure S23 ^{13}C NMR (125MHz, C_5D_5N) spectrum of compound **2b**

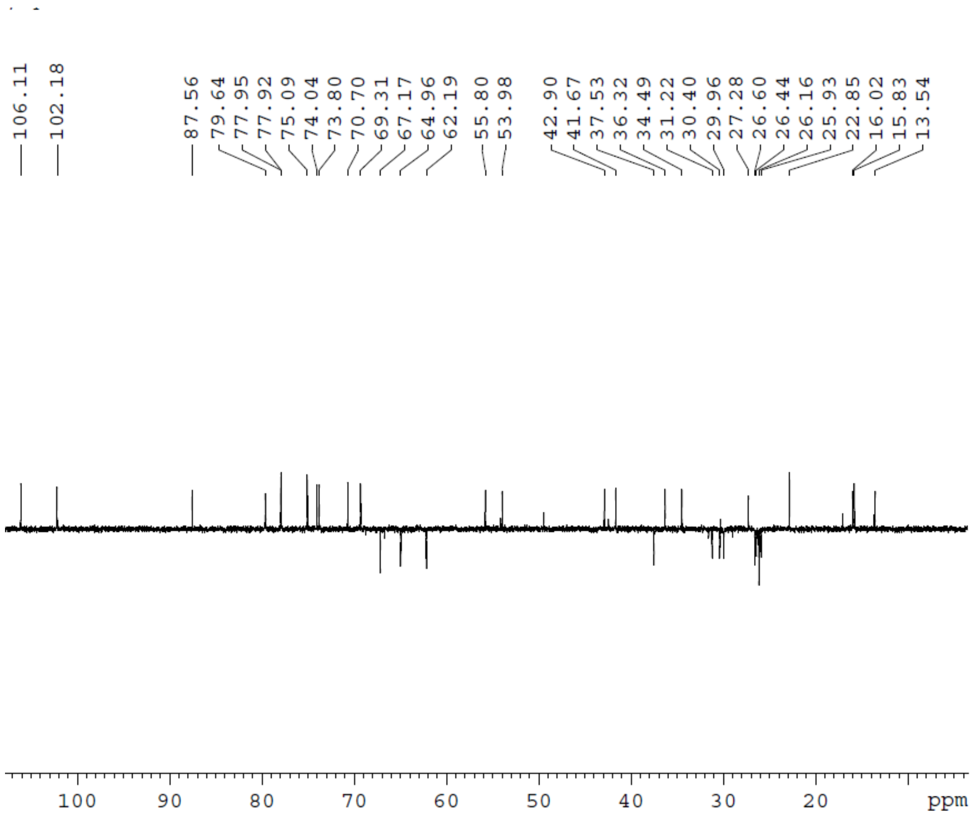

Figure S24 The DEPT 135 (C_5D_5N) spectrum of compound **2b**

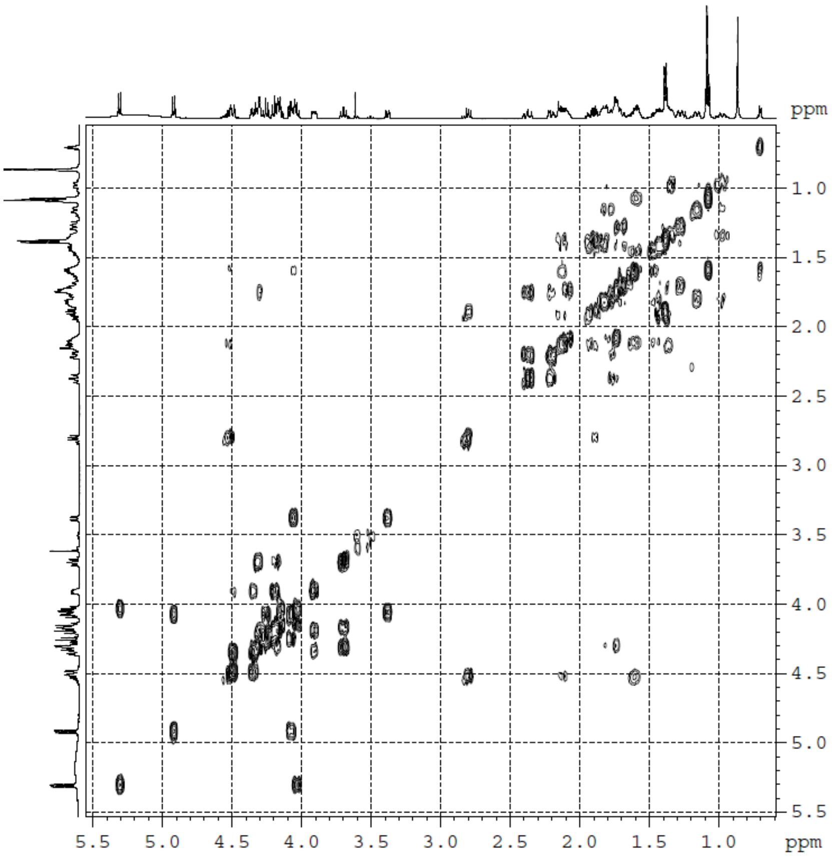

Figure S25 The ^1H ^1H COSY (C_5D_5N) spectrum of compound **2b**

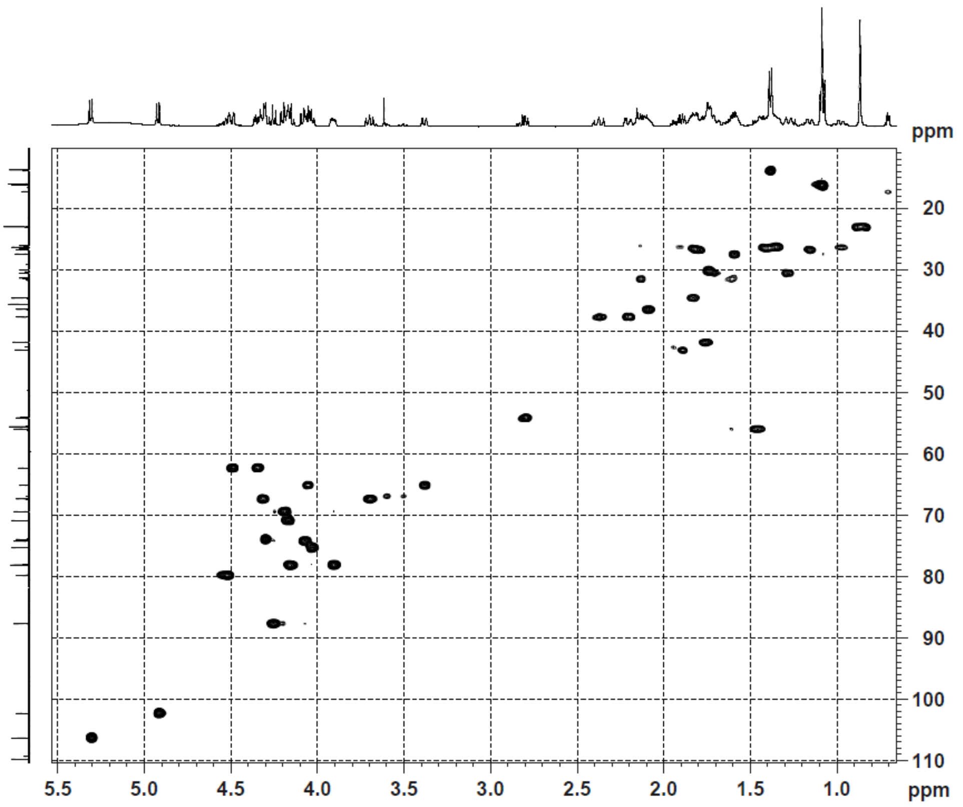

Figure S26 The HSQC (C_5D_5N) spectrum of compound **2b**

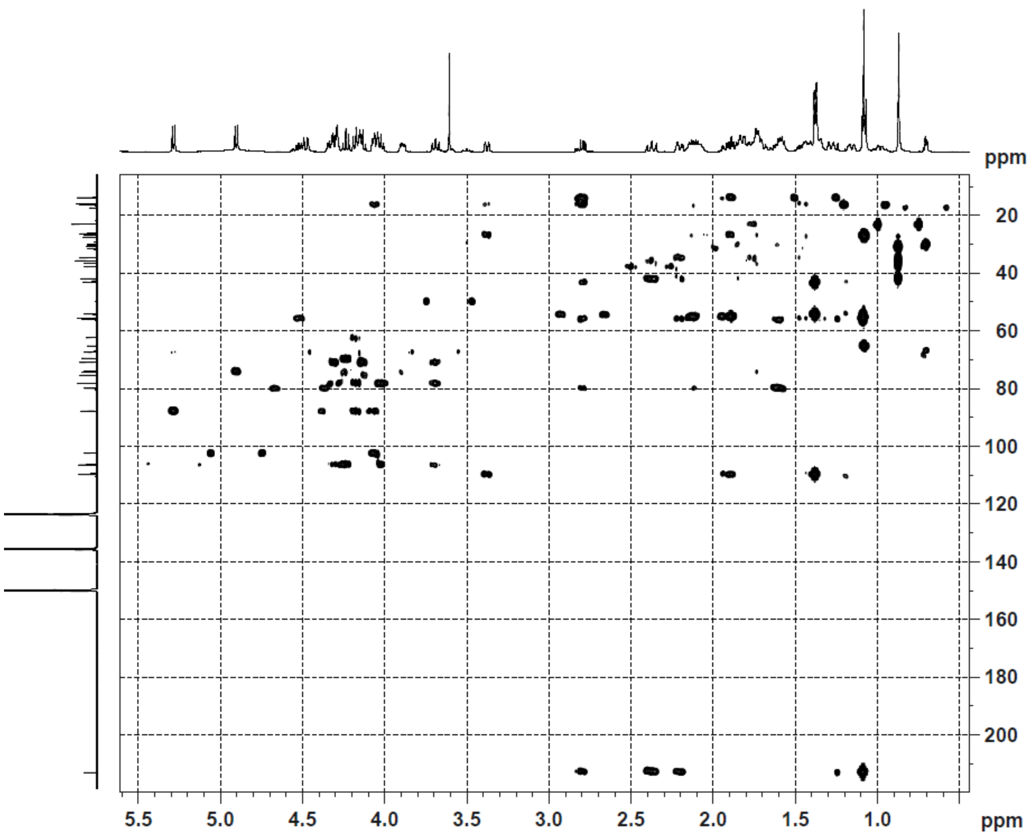

Figure S27 The HMBC (C_5D_5N) spectrum of compound **2b**

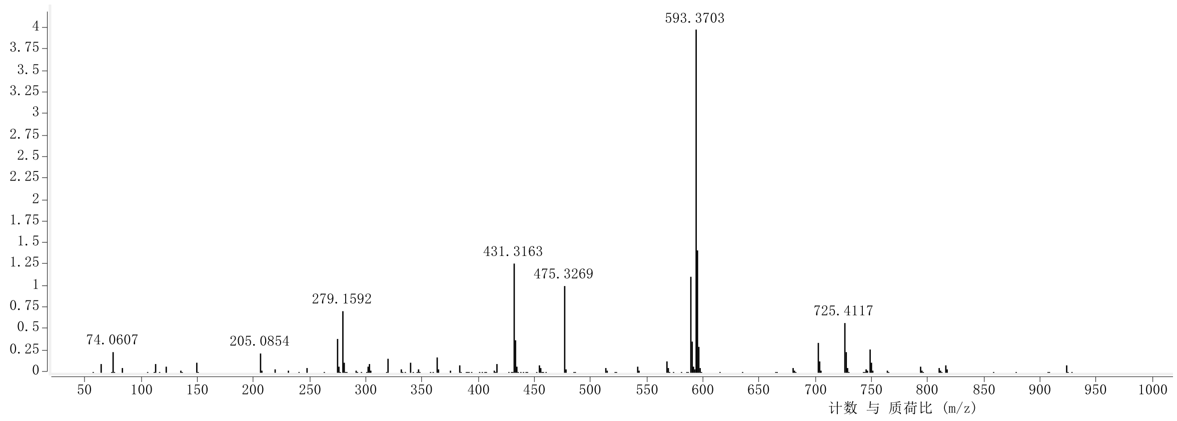

Figure S28 The HRESI-TOF-MS spectrum of compound **2b**

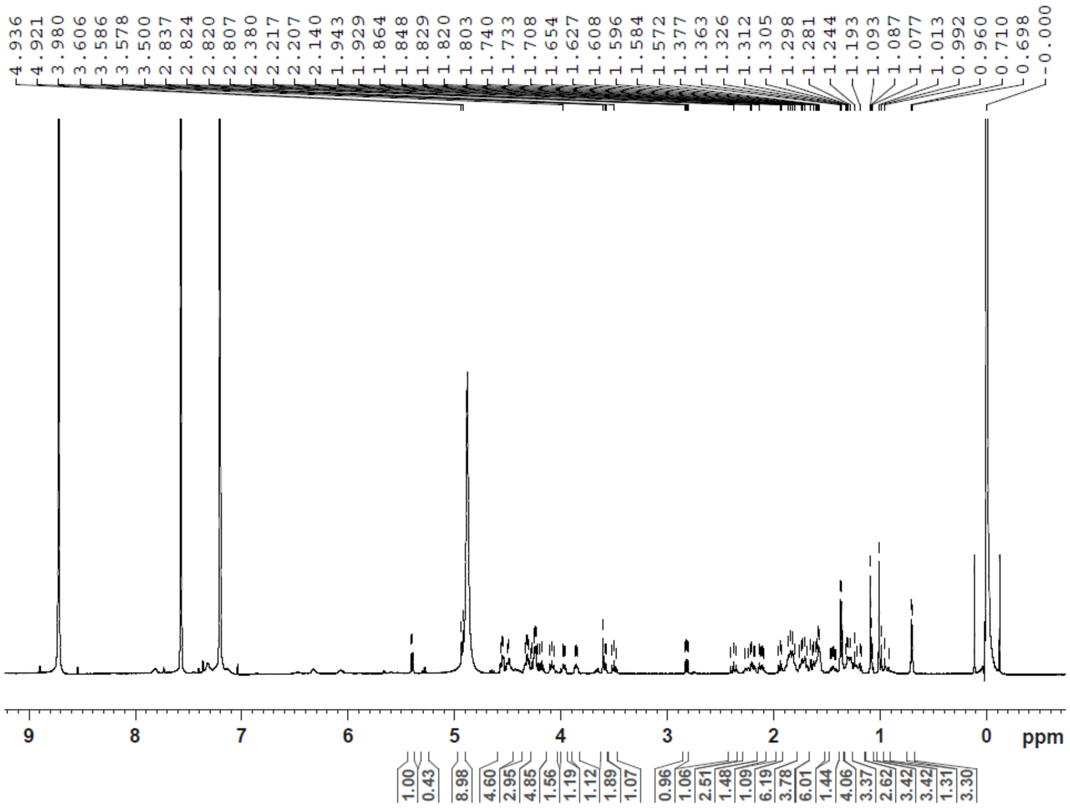

Figure S29 ^{1}H NMR (500MHz, C_5D_5N) spectrum of compound **3a**

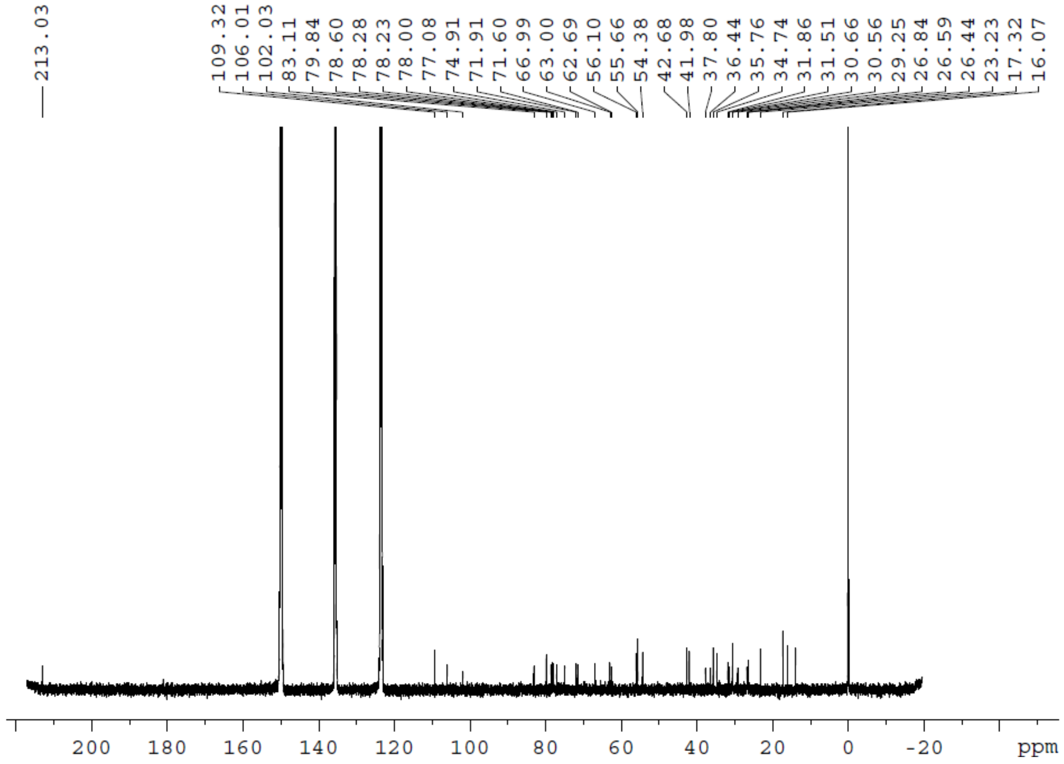

Figure S30 ^{13}C NMR (125MHz, C_5D_5N) spectrum of compound **3a**

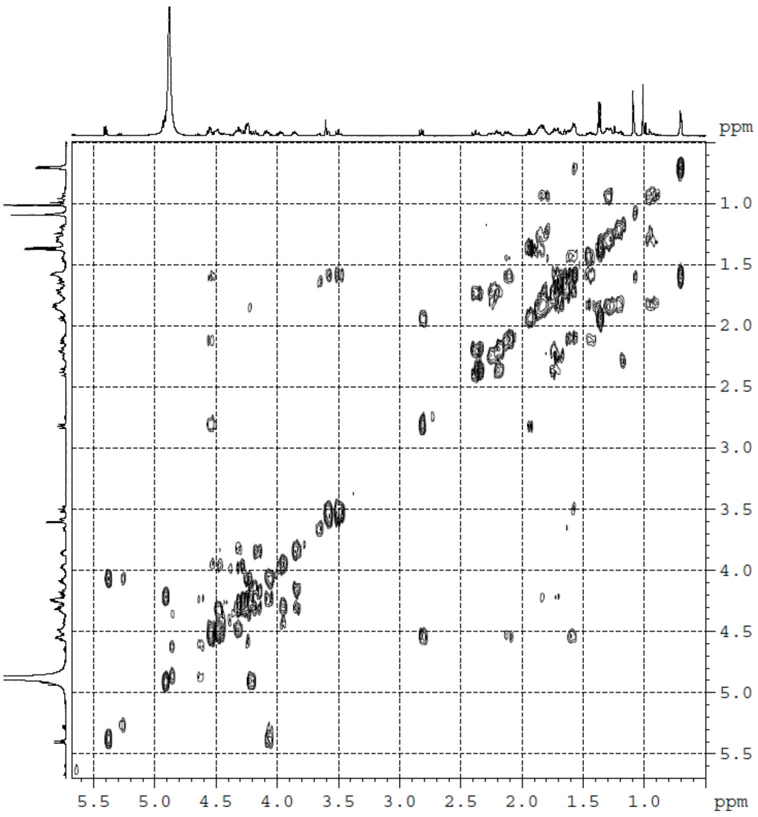

Figure S31 The ^{1}H ^{1}H COSY (C_5D_5N) spectrum of compound **3a**

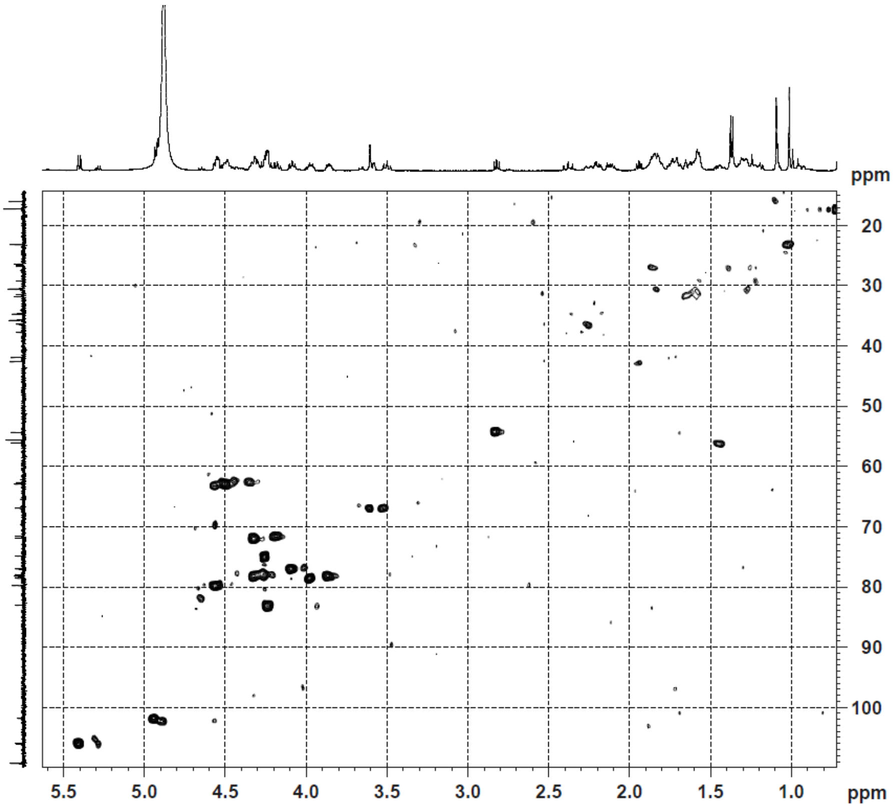

Figure S32 The HSQC (C_5D_5N) spectrum of compound **3a**

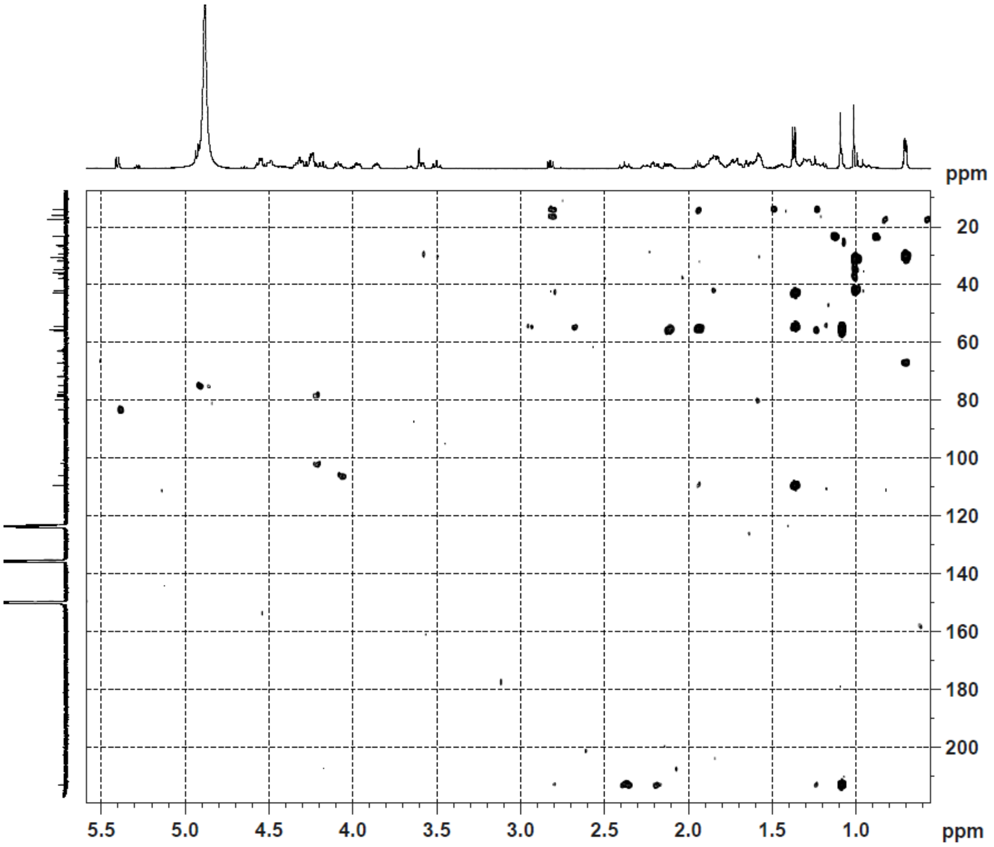

Figure S33 The HMBC (C_5D_5N) spectrum of compound **3a**

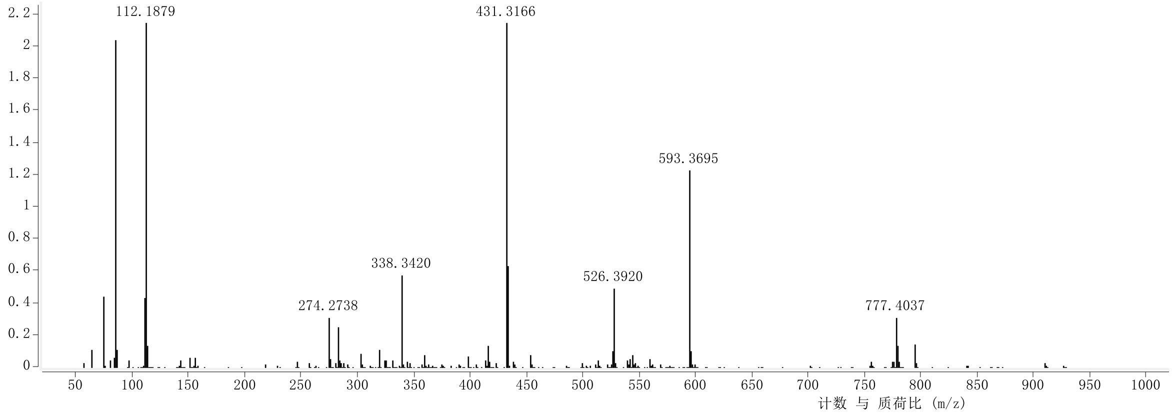

Figure S34 The HRESI-TOF-MS spectrum of compound **3a**

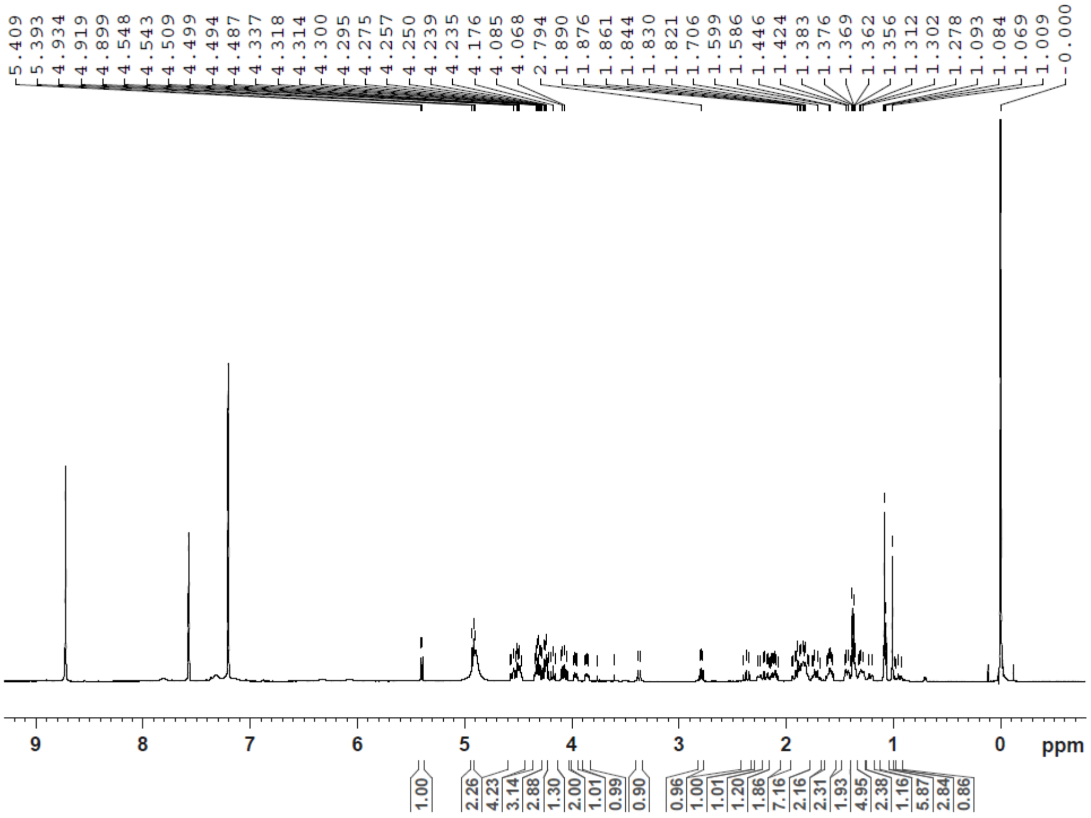

Figure S35 ^{1}H NMR (500MHz, C_5D_5N) spectrum of compound **3b**

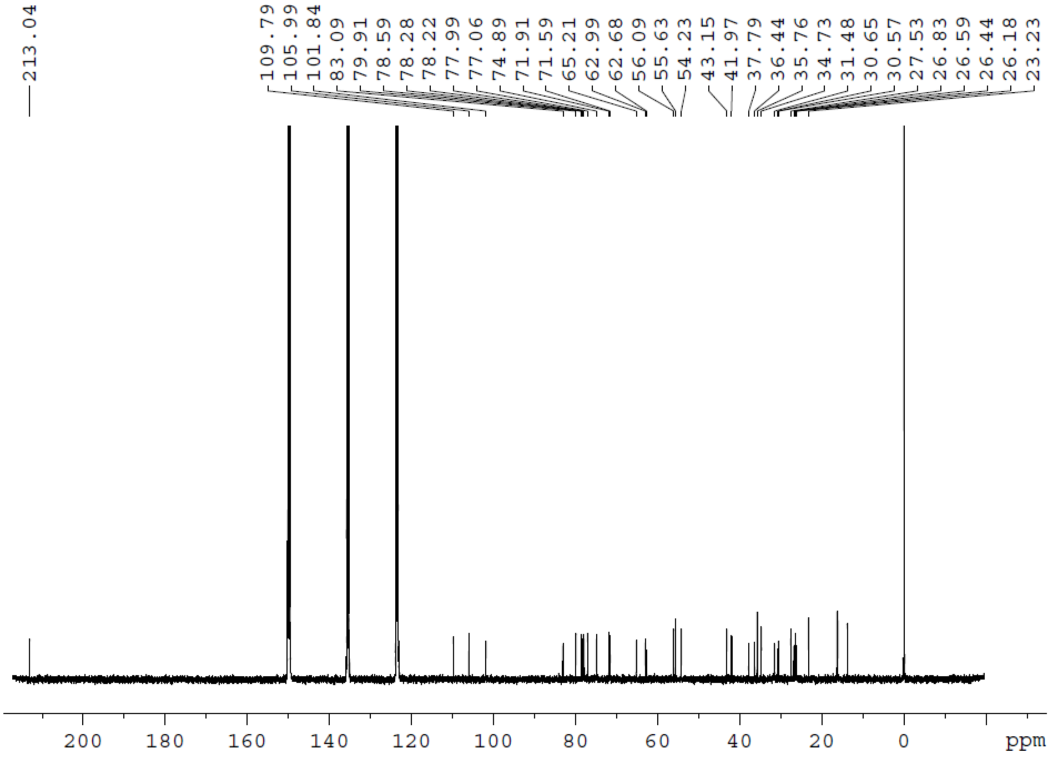

Figure S36 ^{13}C NMR (125MHz, C_5D_5N) spectrum of compound **3b**

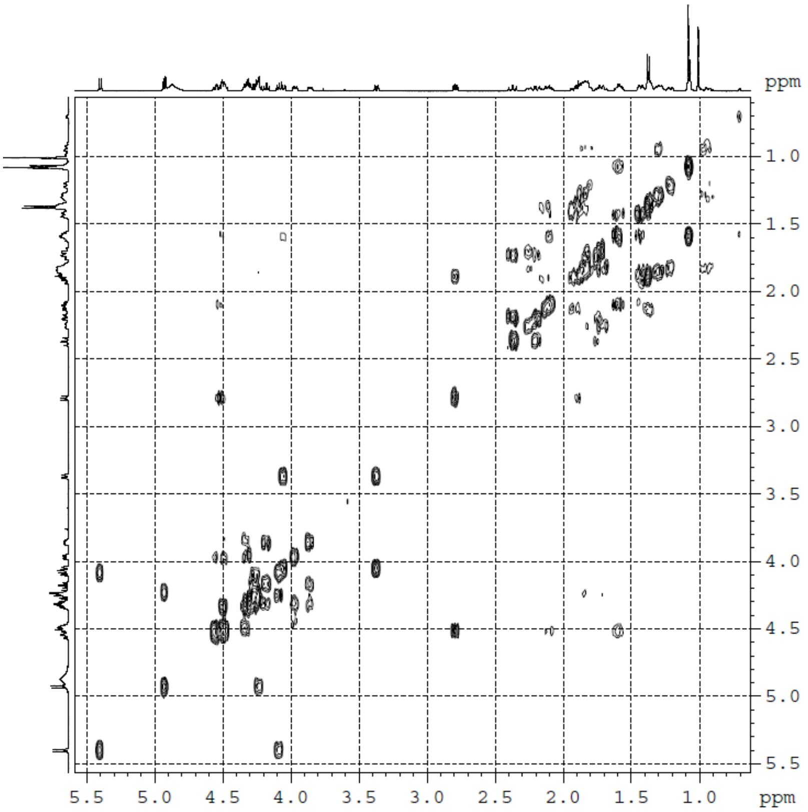

Figure S37 The ^{1}H ^{1}H COSY (C_5D_5N) spectrum of compound **3b**

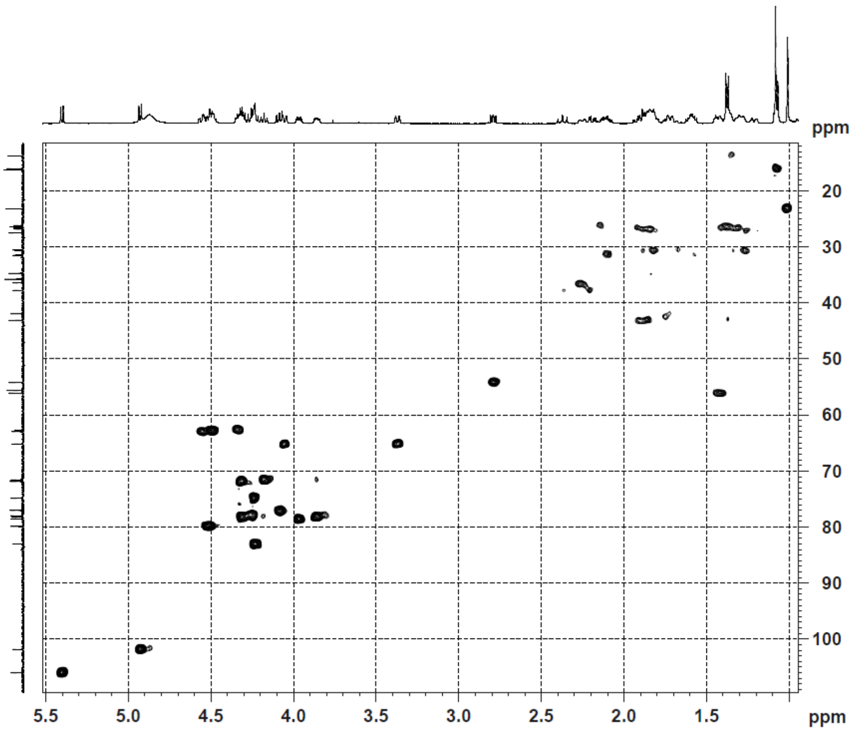

Figure S38 The HSQC (C_5D_5N) spectrum of compound **3b**

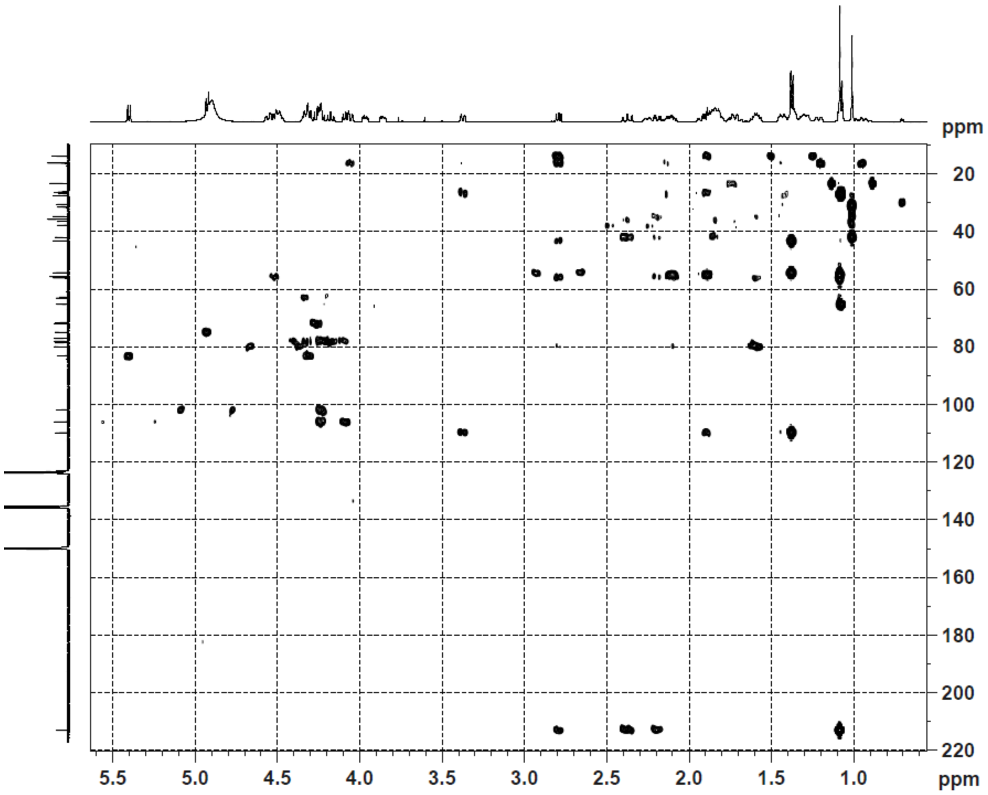

Figure S39 The HMBC (C_5D_5N) spectrum of compound **3b**

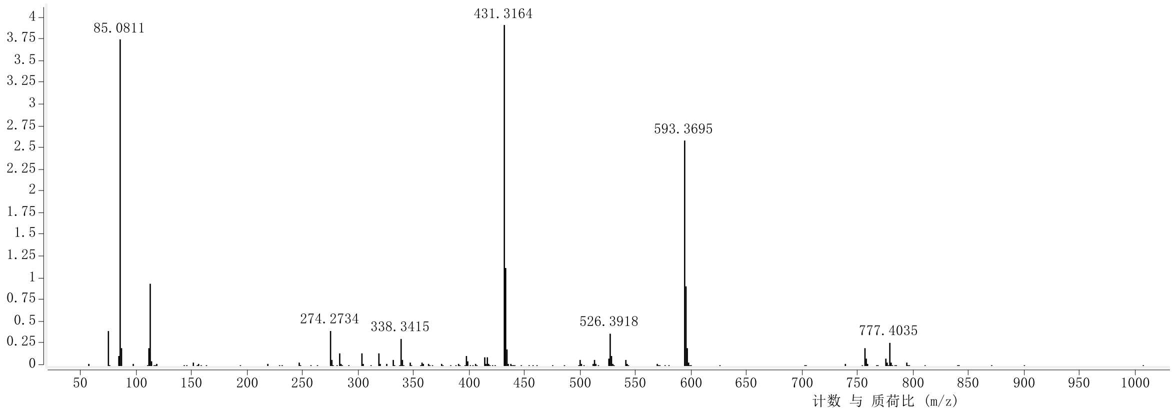

Figure S40 The HRESI-TOF-MS spectrum of compound **3b**
